# Origins of second tumors in children and mutational footprint of chemotherapy in normal tissues

**DOI:** 10.1158/2159-8290.CD-23-1186

**Published:** 2024-06-03

**Authors:** Mònica Sánchez-Guixé, Ferran Muiños, Morena Pinheiro-Santin, Víctor González-Huici, Carlos J. Rodriguez-Hernandez, Alexandra Avgustinova, Cinzia Lavarino, Abel González-Pérez, Jaume Mora, Núria López-Bigas

**Affiliations:** 1 Institute for Research in Biomedicine (IRB Barcelona), The Barcelona Institute of Science and Technology, Baldiri Reixac, 10, 08028 Barcelona, Spain; 2 Centro de Investigación Biomédica en Red en Cáncer (CIBERONC), Instituto de Salud Carlos III, Madrid, Spain; 3 Institut de Recerca Sant Joan de Déu, Barcelona 08950, Spain; 4 Pediatric Cancer Center Barcelona (PCCB). Hospital Sant Joan de Déu. 08950 Barcelona. Spain; 5 Institució Catalana de Recerca i Estudis Avançats (ICREA), Barcelona, Spain; 6 Universitat Pompeu Fabra, Barcelona, Spain

## Abstract

Pediatric cancers are rare diseases, and children without known germline predisposing conditions who develop a second malignancy during developmental ages are extremely rare. We present four such clinical cases and through whole-genome and error-correcting ultra-deep duplex sequencing of tumor and normal samples, we explored the origin of the second malignancy in four children, uncovering different routes of development. The exposure to cytotoxic therapies was linked to the emergence of a secondary AML. A common somatic mutation acquired early during embryonic development was the driver of two solid malignancies in another child. In two cases, the two tumors developed from completely independent clones diverging during embryogenesis. Importantly, we demonstrate that platinum-based therapies contributed at least in one order of magnitude more mutations per day of exposure than aging to normal tissues in these children.

## Introduction

Childhood cancers are all rare diseases, most with some evidence of an embryonal cell of origin(1). In extremely unusual instances, a second tumor presents years after a child is cured of a first primary malignancy. If this second tumor is of a different type and unrelated to the first malignancy, there are several possibilities to explain its origin. It may be related to underlying germline cancer predisposition (e.g., an osteosarcoma developing in an *RB* mutated individual having survived a retinoblastoma previously(2)), or an early mosaic somatic mutation acquired during fetal development(3). Exceptionally, two tumors of the same type have been described to arise independently during embryonic development in a child with pathogenic germline variant(4). Finally, the second malignancy may be caused by the mutagenic and/or cancer promoting effect of the cytotoxic therapies used during the treatment of the first neoplasm (e.g., AML secondary to therapy(5− 7)).

We and others have studied the mutagenic effect of cytotoxic drugs on both tumor and normal cells(6,8− 10). We have shown that the mutagenic effect can be traced through their specific mutational profiles −often referred to as mutational footprints− which may be recognized in the mutations of samples taken after drug exposure. However, identifying chemotherapy induced mutations in normal tissues is hampered by the difficulty of obtaining the samples and the technical challenge of detecting very low variant allele frequency. As a result, despite scattered reports(6,11− 13), we still lack a comprehensive understanding of the contribution of cytotoxic drugs to the mutational burden of normal tissues in cancer survivors. These mutations have been speculated to contribute to the long-term side effects of chemotherapy, although there is yet no conclusive evidence to support this hypothesis. Therefore, understanding the mutagenic effects of chemotherapy is critically important for childhood cancer survivors, who will likely live many years after treatment.

Here, we describe four cases of children who were cured of a primary malignant tumor and were diagnosed with a second malignancy several years later, with no recognizable germline predisposing variants explaining the origin of the second tumors (Supplementary Table S1 and Supplementary Methods). We studied samples from the tumors and normal tissues of these children using whole-genome sequencing and targeted deep duplex DNA sequencing (Supplementary Fig. S1A, B). We found different explanations underlying the development of the four second tumors: the exposure to cytotoxic therapies; a common somatic driver mutation acquired early during embryonic development; or the independent acquisition of two somatic driver events during embryonic development. Across all children exposed to platinum-related therapies, we detected a high mutational burden contributed by these drugs to normal tissues.

## Results

### Acute myeloid leukemia secondary to exposure to cytotoxic therapy

**Figure 1:**
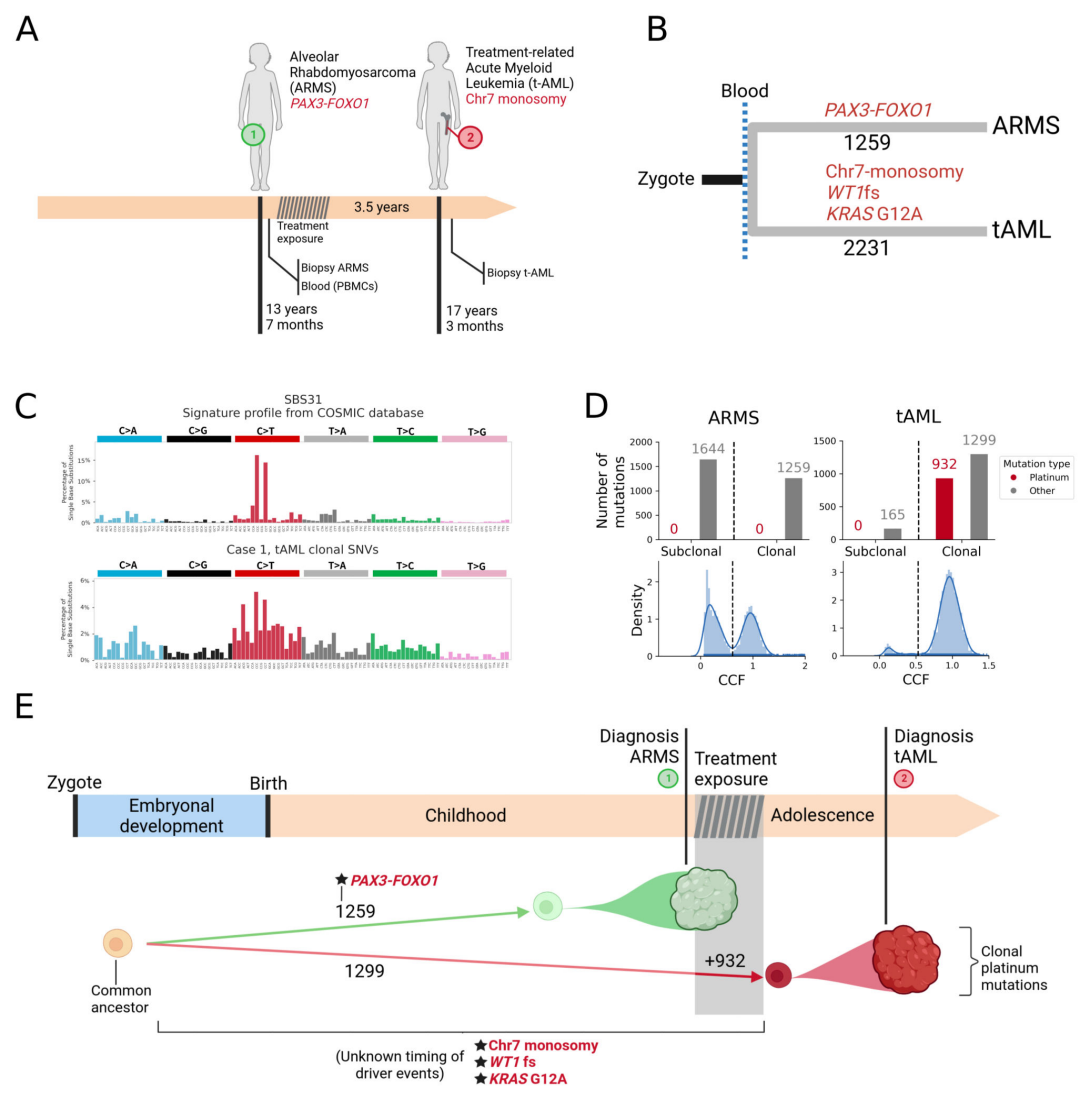
The origin of therapy-related AML of case 1. **A**, Three and a half years after the development of ARMS, case 1 presented with Acute Myeloid Leukemia. **B**, The analysis of whole-genome somatic mutations in both tumors revealed their totally independent origin after the separation of the ARMS and blood lineages (in gastrulation). **C**, Visual comparison of single base substitution signature (SBS) 31 (associated with exposure to platinum-based drugs) and the mutational profile of the tAML reveals common maxima of mutational frequency, suggesting the presence of SBS31 in the tAML. **D**, A careful reconstruction of the activity of SBS31 (and all other mutational signatures) contributing to the mutational profile of the ARMS and tAML yields estimates of the activity of both. SBS31 is active in the clonal mutations of the tAML, while it is absent from the mutations of the ARMS. **E**, Schematic representation of the evolution of the two tumors of case 1.

Case 1 presented with non-metastatic alveolar rhabdomyosarcoma (ARMS) in the right lower extremity at 13 years and 7 months of age. The child received chemotherapy, including irinotecan and carboplatin, subsequent radical surgery and radiotherapy achieving long term complete remission. She had continued normal development, when at 17 years of age presented with a therapy-related Acute Myeloid Leukemia (tAML), a common type of secondary malignancy (Fig. 1A; Supplementary Fig. S1A; case history in Supplementary Information).

We sequenced the whole genome of the ARMS, the tAML and a normal blood sample obtained at the time of the first diagnosis, all at a median 120X depth. Somatic mutations in the ARMS and tAML were identified using the normal blood sample as reference of the germline genome (Methods; Supplementary Fig. S2A) and clonal mutations were identified based on their variant allele frequency (Supplementary Fig. S2B-D). This analysis revealed the presence in the ARMS of the pathognomonic *PAX3-FOXO1* fusion driver gene, resulting from a reciprocal balanced translocation between chromosomes 2 and 13(14,15). The analysis of the tAML yielded as potential drivers an oncogenic *KRAS* G12A missense variant, a *WT1* frameshift indel(16) and a monosomy of chromosome 7(17). Overall, 1259 and 2231 somatic clonal single nucleotide variants (SNVs) were identified in the ARMS and tAML, respectively, with no shared clonal SNVs between the two (Fig. 1B; Supplementary Fig. S3A). This indicated that both malignancies emerged from cells whose lineages separated before or at the time when the hematopoietic tissue arose during fetal development.

Next we asked whether the tAML had begun its expansion from a single hematopoietic stem cell during or after the exposure to chemotherapy as previously described in adults with tAML (6). To answer this question, we and others have previously used the mutational footprints left by chemotherapies employed in the treatment of the primary tumors of cancer patients (6,9,18). The rationale behind this analysis is that while the mutations caused by the DNA-damaging agent used to treat the primary tumor are different in every exposed cell, if a clonal expansion of one of these cells takes place after the exposure −as in the case of a relapse or metastasis−, its mutations (including those left by the chemotherapy) will rise above the limit of detection of bulk sequencing. If this clonal expansion is complete, with all the cells in the new malignancy derived from one cell that was exposed to the treatment, chemotherapy-related mutations will appear as clonal upon sequencing (Supplementary Fig. S2B-D).

While in principle any chemotherapy leaving a discernible mutational footprint could be used for this purpose, platinum-based drugs have been employed before, because they are known to leave a distinct mutational footprint(6,8,9,19). Specifically, we previously reported the presence of platinum-related footprints across clonal mutations in eight adult tAML, indicating that the full clonal expansion from a single hematopoietic stem cell had occurred after drug exposure in all cases(6,18). To ascertain whether the same was true in this pediatric tAML, we analyzed the tri-nucleotide profiles of its clonal and subclonal mutations. Visual inspection of the mutational profiles of the tAML sample revealed a high frequency of C>T nucleotide changes in the C[C>T]T and C[C>T]C tri-nucleotide context (Fig. 1C). The high frequency of these two types of mutations, the most active in SBS31 (and among the most active in SBS35), indicates the presence of platinum-induced mutations in the tAML(6,8,9). To carefully estimate the contribution of platinum-related mutations to the tAML, we asked if the inclusion of SBS31 or SBS35 significantly improved the reconstruction of these profiles, compared to their absence (Methods). We found that SBS31 (or SBS35) contributed to the clonal mutations of the tAML (Fig. 1D and Supplementary Fig. S3B). The presence of platinum-related mutations in all tAML cells (i.e., within clonal mutations) can only be explained if the tAML originated from one hematopoietic stem cell that expanded upon the exposure to platinum (Supplementary Fig. S2B-D; Supplementary Information). As expected, no platinum-related footprint was observed in the ARMS, developed prior to platinum exposure (Fig. 1D). Therefore, as in the case of adult tAMLs, this pediatric tAML experienced full clonal expansion after the exposure to cytotoxic drugs (Fig. 1E).

Finally, we explored the question of whether the driver mutations of the tAML could have been contributed by the exposure to the carboplatin. To that end, we computed the possibility that the *KRAS* driver mutation (the only driver base substitution) was generated by either SBS31 or SBS35. We estimate that this mutation has between 23% (SBS35) and 32% (SBS31) probability of having been generated by the platinum exposure. In comparison, the calculation yielded a 61%-72% probability of having been generated by the age-related SBS5, in which case it could conceivably have been pre-existing to the exposure. In any case, the activity of the platinum mutational footprint among clonal mutations in the tAML implies that a clonal sweep occurred after the start of the treatment, whether its driver mutations were pre-existent or contributed by the cytotoxic therapy. A summary of the somatic mutation landscape of both tumors of case 1 appears in Supplementary Figures 3C and D and Supplementary Table S2.

### Common origin of two solid malignancies

**Figure 2:**
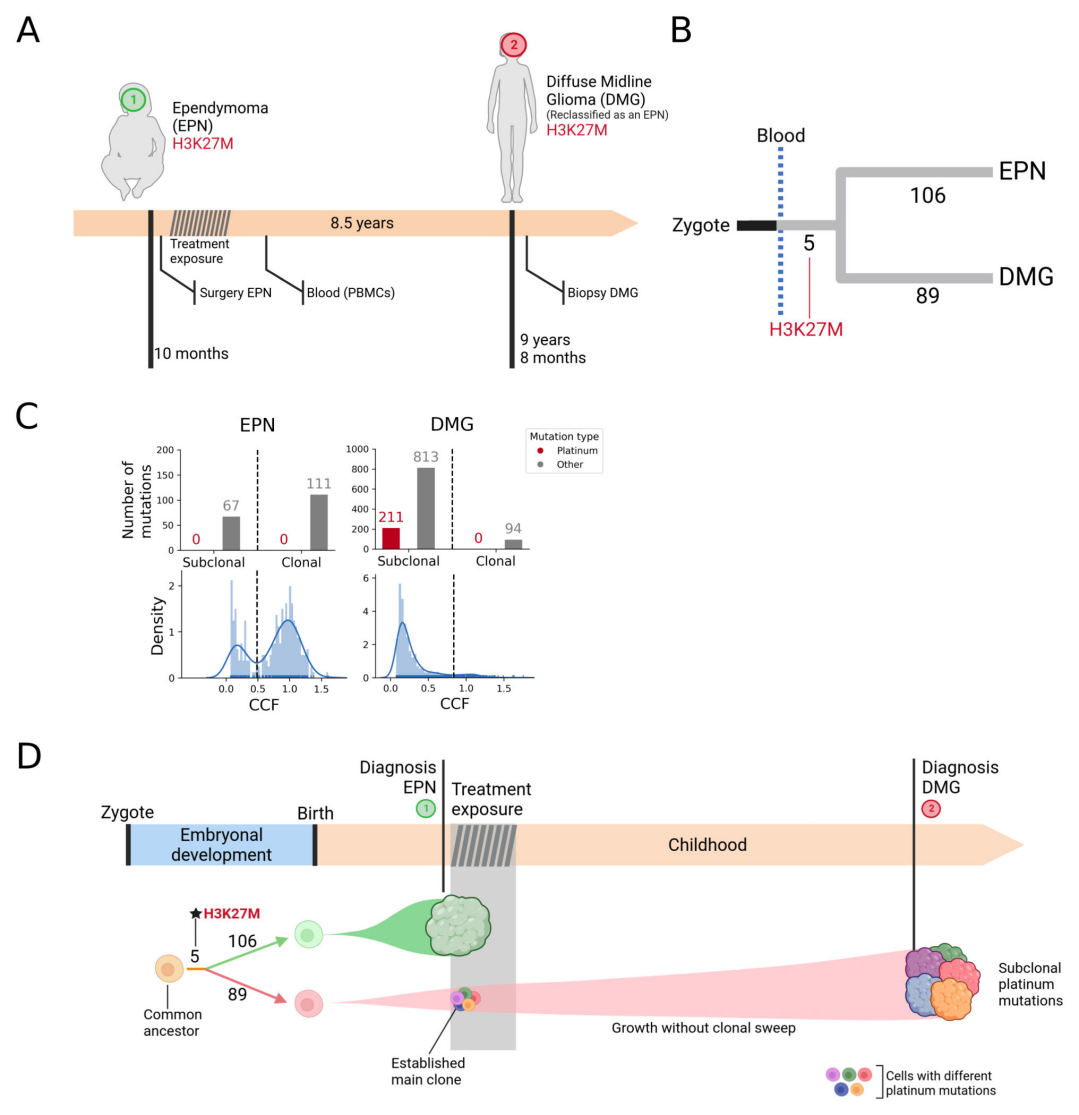
The origin of the second tumor in case 2. **A**, Eight and a half years after the development of EPN, case 2 presented with DMG (subsequently reclassified as an EPN during the preparation of this manuscript). **B**, The analysis of whole-genome somatic mutations in both tumors revealed a common somatic driver mutation followed by completely independent development. **C**, A careful reconstruction of the activity of SBS31 (and all other mutational signatures) contributing to the mutational profile of the EPN and DMG yields estimates of the activity of both. SBS31 appears active only across subclonal mutations of the DMG, revealing that it was already present at the time of diagnosis of the EPN. **D**, Schematic representation of the evolution of the two tumors of case 2.

Case 2 was diagnosed with an anaplastic ependymoma (EPN), WHO grade 3 centered in the IVth ventricle at 10 months of age. The tumor was completely resected and the child received adjuvant chemotherapy with irinotecan and cisplatin and subsequently proton beam radiotherapy for a total dose of 59.4 GY, and was followed closely given the associated bone malformations compatible with Trevor syndrome. After 8.5 years, a brain MRI showed a second tumor centered in the right ponto-bulbar angle, which was biopsied and diagnosed as a H3K27-mutant diffuse midline glioma (DMG), CNS WHO grade 4 (Fig. 2A; Supplementary Fig. S1A and B).

We sequenced the whole-genome of both tumors, and a blood sample at a median 120X depth. Somatic mutations in both tumors were identified using the blood sample as the reference germline genome of the child (Methods; Supplementary Fig. S2A) and clonal mutations were identified based on their variant allele frequency (Supplementary Fig. S2B-D). Five somatic SNVs not present in the reference blood sample were identified in the two tumors, indicating a common origin following the divergence between their shared neural lineage and the hematopoietic tissue (Fig. 2B and Supplementary Fig. S4A). One of these mutations was the K27M SNV in *HIST1H3B*, which had been previously identified as the driver of the DMG (and subsequently found also in the EPN), an uncommon founder event of posterior fossa group A EPN(20− 22). All remaining clonal somatic mutations were unique to each tumor (106 for the EPN and 89 for the DMG), pointing to an independent development after early divergence of the cell lineages that gave rise to both malignancies. Motivated by the presence of the H3K27M mutation in both tumors, we carried out a comparison of their methylomes. This showed that they also shared very similar methylation profiles, consistent with a Posterior Fossa Ependymoma Group A1 (calibrated score >0.9), which would probably result in a reclassification of the DMG as an EPN. Nevertheless, notice that although both tumors are classified as EPN, their development was completely independent, as evidenced by the lack of overlap between their clonal mutations (excluding the five mosaic mutations acquired early in development).

We were intrigued by the mismatch between the similar number of clonal mutations of both tumors and the long time (8.5 years) elapsed between their clinical diagnoses, as well as by the much greater number of subclonal mutations found in the DMG compared with the EPN (Fig. 2C). To explain these facts, we hypothesized that both tumors could have originated contemporaneously, with the DMG remaining clinically undetectable. To test this hypothesis we examined the tri-nucleotide profile of clonal and subclonal mutations identified in the DMG to detect whether the inclusion of platinum-related signatures (SBS31 or SBS35) significantly improved their reconstruction. The platinum-related signatures were found to be active only among the subclonal mutations of the DMG (Fig 2C and Supplementary Fig. S4B). No platinum-related mutations were detected in the sample of the EPN, excised before treatment. If the second tumor had started its expansion from a single cell during platinum exposure, it should bear clonal platinum-related mutations (as in the case of the tAML). Their absence thus implies the absence of a clonal sweep in the evolution of this second tumor after cisplatin exposure, indicating that the DMG was already present as a multicellular entity at the time of the treatment, at 10 months of age. It then continued to accumulate subclonal mutations and slowly expanded for 8.5 years until becoming clinically apparent (Fig. 2D). A summary of the somatic mutation landscape of the two tumors of case 2 is represented in Supplementary Figures 4C and D and Supplementary Table S2.

### Independent origin of two solid malignancies

**Figure 3:**
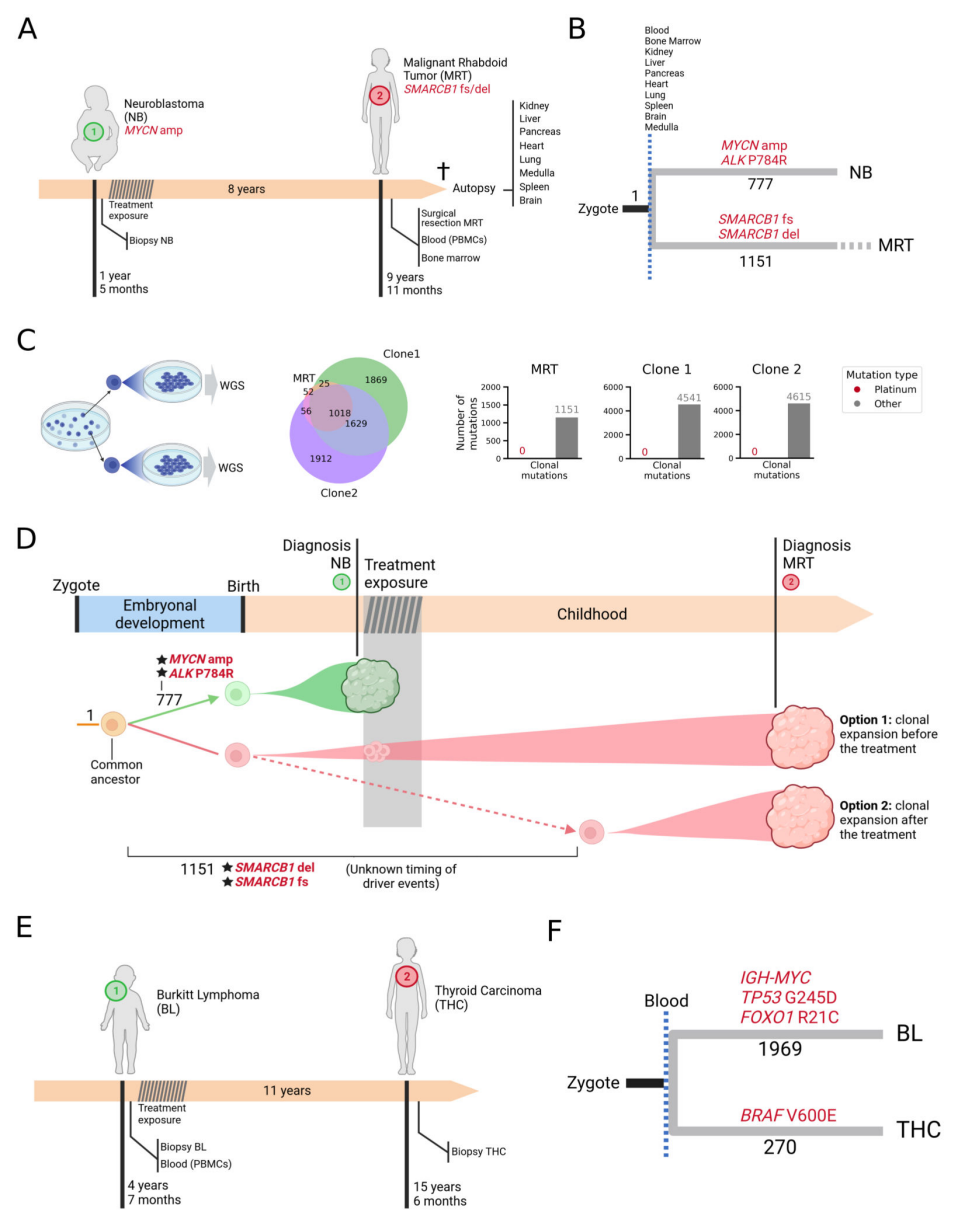
The origin of second solid tumors of cases 3 and 4. **A**, Eight years after the development of NB, case 3 presented with MRT. **B**, The comparison of whole-genome mutations in both tumors with those detected across samples of ten normal tissues (some obtained during autopsy) revealed a completely independent origin of the NB and the MRT. **C**, No platinum-related mutations were detected in expanded single cells of the MRT. Therefore there is apparently no mutagenic contribution of cisplatin to the mutational burden of the MRT. **D**, Schematic representation of the evolution of the two tumors of case 3. **E**, Eleven years after the development of BL, case 4 presented with THC. **F**, The analysis of whole-genome somatic mutations in both tumors revealed a totally independent origin of both tumors.

Case 3 presented at 17 months of age and was diagnosed with *MYCN* amplified, undifferentiated neuroblastoma (NB). The child received treatment for high-risk NB with induction chemotherapy including cisplatin, followed by surgery, radiotherapy, and further cycles of high dose chemotherapy. She achieved complete remission and went on to receive mu3F8-based immunotherapy, finishing 20 months after initial diagnosis. After developing normally for 8.5 years, at age 10, she developed a malignant rhabdoid tumor (MRT) of the soft tissues with biallelic loss of *SMARCB1*. Six months later, the child died of progressive disease. The parents granted the autopsy and tissue samples were preserved by snap-freezing (Fig. 3A; Supplementary Fig. S1A).

We sequenced the whole genome of the primary tumor (NB), the secondary tumor (MRT), as well as blood and bone marrow samples taken at the time of diagnosis of the MRT, and macroscopically normal samples from kidney, liver, pancreas, heart, lung, spinal cord, spleen, and brain obtained at autopsy, all at a median depth of 120X. In addition, we sequenced (~30X) the whole genome of blood samples taken from both parents (Supplementary Fig. S5A). Inherited variants were filtered out through comparison with the parents’ DNA, and *de novo* germline variants were identified as those with variant allele frequency (VAF) around 0.5 or 1 (i.e., in heterozygosis or homozygosis in all cells) of all the individual’s tissues (Supplementary Fig. S5A-C and Supplementary Methods). Clonal somatic SNVs, indels, and larger structural variants in each tumor were confirmed by direct somatic tumor-blood calling (Supplementary Fig. S2A and Supplementary Methods). This corroborated the high level (307 copies) amplification of the *MYCN* locus as driver event of the NB, and identified another potential driver event, a nonsynonymous SNV (P784R) of *ALK*. It also corroborated, in the MRT, the driver bi-allelic inactivation (5 nt frameshift and large 17M bp deletion) of *SMARCB1* (Fig. 3B.

Only one somatic clonal mutation was shared between the NB and the MRT, but this appeared also across all normal tissues at low VAF, (Fig. 3B and Supplementary Fig. S5D), indicating that it could be germline *de novo* or a very early mosaic somatic mutation. Thus, as described in case 1, the lineages that gave rise to the founder cells of the NB and the MRT diverged very early in development.

In an effort to time the onset of the clonal expansion of the MRT, we resorted to the platinum mutational footprint. Surprisingly, we found no trace of SBS31 or SBS35 across MRT clonal or subclonal mutations (Supplementary Fig. S6A). Nevertheless, platinum-related mutations could still be present below the limit of detection of whole-genome sequencing of the bulk tumor, if the MRT clone was already established at that time (see above), and it did not experience a clonal sweep after the exposure to cisplatin. To test this possibility, we carried out error-correcting high-depth (~20.000X) targeted duplex DNA sequencing of the MRT. Furthermore, we analyzed whole-genome mutations identified in two cell lines obtained from clonally expanded single cells from the MRT. Still, these two experiments yielded no trace of platinum-related mutations (Fig. 3C and Supplementary Fig. S6B and S6C). Thus, we are unable to precisely determine the time of emergence of the MRT (Fig. 3D). Moreover, the lack of platinum-related mutations in the MRT presents as an unexpected result. We hypothesize that quiescent rhabdoid cells may have developed in a niche protected from cisplatin exposure. The existence of stem cells protected from smoking-related damage has been recently proposed to explain the finding of bronchial cells with unexpectedly few tobacco-related mutations in former smokers(23).

We were able to detect 1073 clonal SNVs and 145 indels of the MRT at low VAF across 10 normal tissues (Supplementary Fig. S5D). We reasoned that this could be explained by the presence across these normal tissues of infiltrating metastatic MRT cells. This was corroborated by the detection by digital PCR of reads supporting the two *SMARCB1* mutations (Supplementary Fig. S7A-D) in histologically normal tissue from kidney, heart, liver and pancreas (Supplementary Fig. S8A), and further confirmed by INI1 immunohistochemical analysis(24) of autopsy samples from the same tissues (Supplementary Fig. S8B). A summary of the somatic mutation landscape of the two tumors of case 3 is represented in Supplementary Figures 9A and B and Supplementary Table S2.

Case 4 was diagnosed with stage 4 Burkitt’s lymphoma (BL) at 4 years of age. After chemotherapy treatment and rituximab, according to the International NHL B 2004 protocol, the child achieved complete remission, without the need of surgery or radiotherapy and did well until a thyroid carcinoma (THC; Fig. 3E) was diagnosed at 15 years of age. Analysis of somatic SNVs and translocations in the BL and THC (identified by direct comparison of 120X median depth whole-genome sequence of both tumors with a reference blood sample) revealed the somatic driver mutations of both tumors (Fig. 3F and Supplementary Fig. S10A). The typical BL *IGH*-*MYC* translocation, a *FOXO1* activating SNV (R21C), and a potential *TP53* loss-of-function SNV were detected in the BL(25,26). A *BRAF* (V600E) SNV −less frequent in children than fusions involving the RET proto-oncogene(27)− was identified in the THC. While 1969 clonal somatic SNVs were identified in the BL, a high mutation burden typical of a germinal center-derived malignancy(28), (Supplementary Fig. S10B,C; Supplementary Table S2) there were only 270 in the THC −a tumor type with a relatively low mutation burden(29). As described in case 3, both tumors shared no somatic SNVs, indicating the early divergence of their cell lineages and their independent origin. Since no platinum-based drugs were used for the treatment of the BL, we were unable to use its mutational footprint to time the emergence of the THC.

### Mutagenic effect of platinum-based therapies on normal tissues

**Figure 4:**
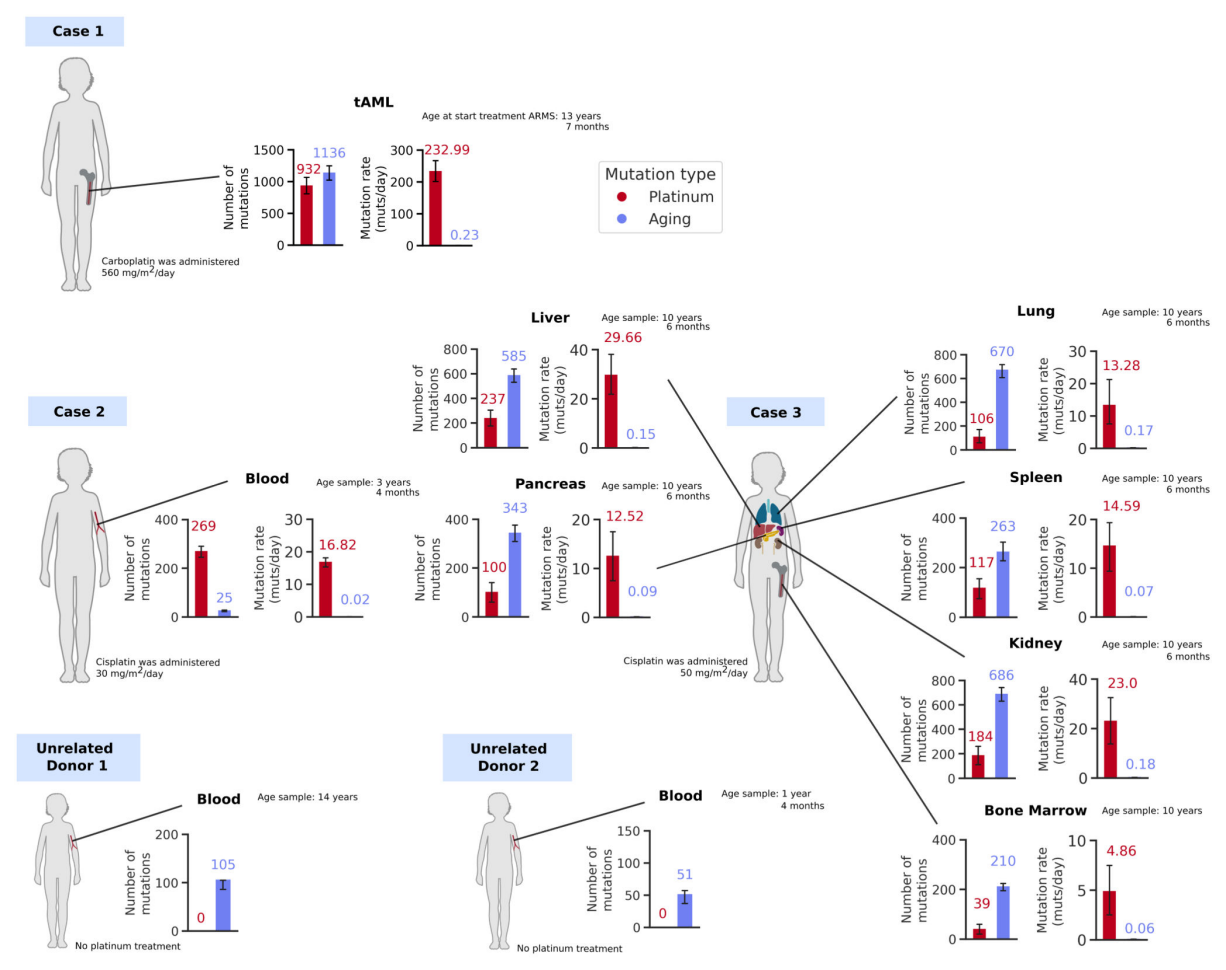
Activity of platinum-related mutational signature across normal tissues of three children. Every pair of bar plots presents the careful deconstruction of the mutational profile of a sample obtained from a tissue (that was normal at the time of exposure to the chemotherapy). Blood samples obtained from unrelated donors 1 and 2 were not exposed to platinum-related drugs. In all cases, the left-hand bars represent the absolute activity of the platinum-based SBS31 (red) and age-related mutational processes (SBS1, SBS5 and SBS40; blue). The rate of age-related mutations of case 1 (tAML) were calculated on the basis of the age of diagnosis of the ARMS, which is an underestimation, since we know its clonal expansion started after its exposure to the treatment. The right-hand bars represent the rate of mutations per day of exposure of both types of mutational processes. In the case of unrelated donors 1 and 2, where no mutagenic platinum activity is detected, only the absolute set of bars is shown.

An important and unexplored question in cancer genomics is the mutagenic effect of cytotoxic therapies in the normal tissues of cancer patients. One of the hurdles for such systematic studies −in particular among children− is the difficulty to obtain samples from different normal tissues across individuals. Usually, these only become available upon autopsy. The second issue is the technical challenge to reliably identify mutations that appear at very low frequency in tissues. One way to bypass this problem, as explained for case 1, is the study of the mutational profile of the tAML, which originated from a hematopoietic stem cell that was not malignant at the time of exposure(6). In this case, we observed 932 clonal (present in the most recent common ancestor of this malignancy) platinum-related mutations. In comparison, 1136 age-related mutations accumulated in this cell before its clonal expansion began, which occurred at some point between the ages of 13 (start of the treatment containing carboplatin) and 17 years (diagnosis of the tAML; Fig. 4). Since the carboplatin treatment comprised 4 days, we can calculate a rate of 232.99 platinum-related mutations accumulated per day of treatment, compared to 0.18 age-related mutations accumulated per day of life.

However, this approach cannot be applied to normal tissues in the absence of a clonal expansion. As we showed above, this hurdle can be overcome using error-correcting high-depth duplex sequencing(30). Thus, we next employed (~20.000X) targeted duplex DNA sequencing to explore the presence of the platinum mutational footprint (SBS31 or SBS35) in blood (case 2) and normal bone marrow (case 3) samples obtained during treatment, as well as normal kidney, liver, pancreas, lung and spleen samples obtained upon autopsy from case 3. The targeted sequencing (mutagenesis) panel employed comprises genomic regions distributed across chromosomes, with a tri-nucleotide content similar to that of the whole human genome(31).

As in the case of the tAML profile of case 1 (Fig. 1C), the normal blood sample from case 2, and the normal liver, kidney and pancreas samples from case 3 exhibited a high frequency of C>T nucleotide changes in the C[C>T]T and C[C>T]C tri-nucleotide contexts (Supplementary Fig. S11), indicative of the presence of platinum-induced mutations in these samples. Careful deconstruction of the mutational profile(6,32) observed across normal tissues obtained from seven organs of both children revealed the presence of platinum-related mutations (Fig. 4; Supplementary Fig. S12A,B; Supplementary Fig. S13; Supplementary Fig. S14). Conversely, no activity was detected in blood samples taken from two unrelated donors unexposed to platinum (Supplementary Fig. S12A,B).

From the number of platinum-related mutations observed throughout the genomic regions covered by the mutagenesis panel, and the depth of duplex sequencing reached at each genomic position, we estimated the total number of platinum-related mutations across the whole genome of a cell (Supplementary Methods). According to this estimation, the number of platinum-related mutations calculated from duplex sequencing of normal tissues is comparable to that observed across all cells of a clonally expanded tissue, such as the tAML. We thus uncovered that the contribution of platinum-based drugs to the mutational burden in normal tissues of cases 2 and 3 ranges from 68 (bone marrow, case 3) to 388 mutations (liver, case 3) per genome (Supplementary Methods; Fig. 4). This represents ~5 to ~30 platinum mutations per day of exposure to the chemotherapy, up to two orders of magnitude higher than the age-related rate of accumulation of mutations in these same tissues.

## Discussion

Here, we analyzed the origin and development of a second malignancy in four children. The four cases have in common the diagnosis of a first malignant solid tumor during infancy, childhood or adolescence, and the development of a second, unrelated malignant tumor 4-11 years later, without any recognizable germline predisposing condition (Supplementary Table S1 and Supplementary Methods). It is important to highlight that the absence of any known predisposing variant does not completely rule out the presence of yet unknown susceptibility germline alteration.

Employing the detection of chemotherapy-related mutational footprints as a tool to time the clonal expansion of the second tumor, we were able to time the clonal expansion of the tAML relative to the exposure. Specifically, the detection of the salient mutational profile of platinum-based drugs (in this case, carboplatin; see history in Supplementary Information) among the clonal mutations of the tAML allowed us to determine that it expanded from its most recent common ancestor upon exposure to chemotherapy, as previously described in adults (6). Therefore, the tAML is the only case of true secondary tumor in our study(33). The role of the platinum chemotherapy might be to supply the mutations driving the clonal expansion of the tAML (see below), to promote the expansion of a previously mutated hematopoietic stem cell, or both(34).

While the exposure to cytotoxic therapies or radiotherapy has been recognized as a cause for the emergence of secondary (mostly hematopoietic) malignancies, their potential role in promoting the development of a pre-existing second solid tumor has −to our knowledge− not been explored before. We found, in case 2, that the H3K27M mutation driving both malignancies appeared shortly after gastrulation, and both tumors had a roughly contemporaneous origin. We can speculate that H3K27M-mutant cells arising from this lineage subsequently expanded into these two brain tumors with the expression profile of each determined by their position in hindbrain-related structures(21). In this case the fact that the second tumor was already present at the time of the primary tumor treatment, and the absence of any subsequent clonal sweep, rules out a role of chemotherapy (or subsequently administered radiotherapy) in its origin. Conversely, in cases 3 and 4, the cells that gave rise to the first and the second tumor originated from cell lineages that separated very early in development, with driver events acquired separately for each tumor. While previous reports have shown that two tumors of the same type may arise independently in one child, frequently due to an inherited cancer predisposition(4), this is, to our knowledge, the first time that a completely independent prenatal somatic origin −i.e., with no obvious germline predisposition (Supplementary Table S1)− has been demonstrated for two different types of rare childhood cancers. In both cases, however, we were unable to time the emergence of the second tumor −in case 3 due to the unexpected absence of platinum related mutations in the MRT, and in case 4, due to lack of exposure to platinum-based therapies in the treatment of the primary tumor.

Of note, while we have used platinum-related mutations to time the emergence of the second tumor relative to the exposure to the therapy, this does not provide any evidence of the potential causative role of cytotoxic therapies in the emergence of the second tumors in any of these cases. In fact, the trinucleotide context A[C>G]C of the KRAS driver mutation in the tAML is more likely to emerge as a mutation by the endogenous SBS5 mutational process than by the platinum mutational footprint (see Supplementary Data). In any case, the presence of the platinum mutational footprint within the clonal mutations of the tAML indicates that a clonal sweep occurred after the exposure to cytotoxic therapy.

Nevertheless, it is intriguing that, in these three children (cases 2, 3 and 4), the time elapsed between the diagnosis of the first and second tumors is longer than that observed in therapy-related myeloid neoplasms in children and adolescents(35). In particular, in case 2, where we demonstrated that the clone giving rise to the DMG was present at the time of the EPN diagnosis, but became clinically visible only after 8.5 years, this invites the question of whether the evolution of the second tumor might have been influenced by the treatment. Similarly, in case 3, the NB developed at 17 months of age, before the median age of presentation of rhabdoid tumors (2 years)(36). Hypothetically, the cytotoxic therapies employed for its treatment could have depleted during infancy the normal growth stimuli necessary for *SMARCB1*-null cells to fully develop into an MRT. As the diagnosis of this tumor coincided with incipient puberty, it is tempting to speculate that the associated changes in systemic growth stimuli facilitated the manifestation of these indolent *SMARCB1*-null cells(37). Indeed, the form of presentation of MRT is statistically related with age. Children with extra-renal non-cranial sites MRT are significantly older than those with renal and CNS rhabdoid tumors(36).

Chemotherapy treatment is known to cause late side effects in childhood cancer survivors(38). The accumulation of mutations caused by the drugs across healthy tissues of the child may be implicated in these late side-effects. With the objective to understand this, we and others previously identified the footprint of some chemotherapies, such as platinum based-drugs in secondary malignancies(6) and, anecdotally, also across some normal tissues (11,12). However, a comprehensive analysis of the mutagenic effects across a broad range of normal tissues has not yet been undertaken. Here, we identified a pervasive mutagenic impact of platinum-based drugs in several normal tissues in all children exposed. To our knowledge this is the first time that chemotherapy-related mutations in normal tissues with very different rates of proliferation have been shown. Up to 100 fold more mutations were contributed by platinum per day of exposure than by endogenous age-related processes. In summary, we show that chemotherapy-related mutations accumulate in healthy tissues, opening up the possibility that they might indeed have long-term implications for the health of childhood cancer survivors.

## Methods

### Sample collection and processing

A sample of the ARMS of case 1, and a matched normal blood sample were collected at diagnosis, and a bone marrow aspirate was collected at the time of tAML diagnosis. A sample of the EPN of case 2 was collected at the time of its resection, and samples of the DMG and matched normal blood were collected at the time of diagnosis. Samples of normal and tumor tissues from case 3 were collected at different times (Fig. 2A), with the NB sample collected at diagnosis, and MRT, matched normal blood and bone marrow aspirate samples collected at the time of MRT diagnosis. Samples of normal (according to H&E histological analysis) kidney, liver, pancreas, heart, lung, medulla, brain and spleen were collected at autopsy. Normal blood samples from both parents of case 3 were also collected. A sample of the BL of case 4 was collected at diagnosis; a sample of the THC was collected after thyroidectomy, and the matched normal blood sample was collected after the diagnosis of the THC. Normal blood samples from two unrelated non-treated pediatric donors were also obtained. DNA from all the samples was extracted using Gentra Puregene kit from Qiagen following the indications from the manufacturer. Library preparation was performed using KAPA HyperPrep Kits.

### Whole-genome DNA bulk sequencing and genomic analysis

Whole genome sequencing was performed from the tumor and matched normal blood samples (and samples of normal tissues of case 3) at a depth of 120X (Supplementary Fig. S2A and S5A). The whole genomes of the blood samples obtained from the parents of case 3 were also sequenced at depth of 30X (Supplementary Fig. S5A). Mutations were called using the matched normal samples as reference in each case: see details in Supplementary Methods.

### DNA methylation profiling

A total of 500 ng of genomic DNA was bisulfite converted using the Zymo EZ DNA Methylation Kit (Zymo Research Irvine), followed by purification with Zymo DNA Clean Kit (Zymo Research Irvine). DNA was hybridized to the Illumina Infinium Methylation EPIC v2.0 BeadChip (Illumina, San Diego). Array intensities were read on an iScan system (Illumina San Diego). Methylation data from the Illumina Infinium Methylation EPIC v2.0 BeadChip were uploaded to the Brain tumor classifier available at www.molecularneuropathology.org for methylation-based classification and chromosomal copy number profile plot.

### Detection of SMARCB1-null variants with digital PCR in case 3

To probe the extent of the presence of the SMARCB1 variants across all 10 normal tissues in case 3, we designed two digital PCR (Supplementary Fig. S8A) probes targeting the two chimeric sequences (for the 5 nt deletion (p.279delVG_V): CCATGTGGGAAACAT as the WT sequence -VIC reporter- and CTGAACATCCATAAACAT as the altered/deleted sequence -FAM reporter-; for the 17 MB deletion: ATGTCTCCACCCTCTCCC as the WT sequence -FAM reporter-, and CTGGCCTGGAAGAGTTTTATAT as the altered/breakpoint sequence -VIC reporter-) (39).

### Detection of SMARCB1-null cells with INI1 immunohistochemistry in case 3

The presence of MRT cells across normal tissues of case 3 was confirmed by immunohistochemistry analysis of the INI1 protein (Purified Mouse Anti-BAF47 Clone 25/BAF47 diluted 1/100, Catalog No: 612110, BD Transduction Laboratories) (Supplementary Fig. S8B).

### Duplex DNA bulk sequencing

In order to explore the mutagenic effect of platinum treatment across normal tissues, we used error-correcting duplex sequencing of the EPN, DMG and normal blood samples of case 2 and the MRT, kidney, liver, pancreas, lung, spleen and bone marrow samples of case 3. DNA library preparation for duplex sequencing followed the protocol described in ref(30). Error-correcting duplex DNA sequencing is based on principles described in refs (30,31).

Purified gDNA (obtained as explained in the previous section) in TE-low buffer was quantified using a Qubit HS DNA kit and 800 ng were used to construct Illumina libraries for Duplex Sequencing using TwinStrand™ reagents and following its protocol. Briefly, after taking the volume to 26 μl, samples were delivered to a 96-well plate, and 14 μl of Fragmentation Buffer 1 and 4 μl of Enzyme Mix per sample added. Fragmentation reaction was carried out in a thermocycler at 25°C for 40 min, and stopped for 20 min at 65°C. Then the end repair and A-tailing reaction (ERAT) was performed, by adding 20 μl of ERAT mix and 1 μl of Fragmentation buffer 2, incubating for 30 min at 20°C and then 30 min at 65°C. After this, ligation of the adapters containing the 8 bp DuplexSeq™ tags was carried out by adding 6 μl of the adapter mix together with 30 μl of the Ligation Reaction Mix, and incubating for 60 min at 20°C. DNA was then purified using SPRI beads (0.8x ratio). Following a conditioning reaction for 60 min at 37°C a PCR was performed to add the sample indexes (45 s at 98°C, then 10 cycles of 15 s at 98°C, 90 s at 60°C and 45 s at 72°C, followed by a 1 min final elongation step at 72°C); DNA was cleaned using SPRI beads (1.0x ratio) and measured by Qubit, obtaining yields between 5.8 and 7.8 μg of total DNA.

A hybridization-capture was then performed o/n (10 min at 95°C; 16 h at 65°C) using the commercial Twinstrand Mutagenesis panel™, with a total size of 48 kb and covering 20 target intervals, representative of genomic regions not believed to be under significant positive or negative selection. Next morning streptavidin beads were added to the reaction, which followed at 65°C for 45 additional minutes. Using a magnet, beads-bound DNA was separated from the supernatant, washed and subjected to in-bead PCR with P5/P7 primers (45 s at 98°C, then 16 cycles of 15 s at 98°C, 45 s at 60°C and 45 s at 72°C, followed by a 1 min final elongation step at 72°C). DNA was cleaned with SPRI beads as described above, and quantified using a Qubit HS DNA kit, with yields between 1.5 and 1.9 μg of DNA. The size distribution as determined by Tapestation, was very homogeneous, between 417-445 bp. Details of the duplex sequencing mutation calling appear in Supplementary Methods.

### Mutational signature analysis

Mutational processes were analyzed using the mutational signature fitting tool mSigAct(32). This tool tests whether the fitting of a sample’s mutational profile improves significantly by the addition of a new signature. In this case, in every sample we tested whether the platinum-related mutational signatures SBS31 and SBS35(8,9) improved the fitting of the mutational profile. The mSigAct provides a p-value from a likelihood ratio test comparing both reconstructed profiles. We developed a synthetic sample-based approach to precisely calculate the impact of SBS31 and SBS35 signatures on each sample: see details of the analysis in Supplementary Methods.

## Supplementary Material

Figure S1

Figure S2

Figure S3

Figure S4

Figure S5

Figure S6

Figure S7

Figure S8

Figure S9

Figure S10

Figure S11

Figure S12

Figure S13

Figure S14

Supplementary information

Table S1

Table S2

## Data Availability

Raw sequencing data (cram files) generated in this analysis for the samples of the 4 cases are deposited in the European Genome-Phenome Archive (EGA) repository (EGAD50000000237, EGAD50000000238, EGAD50000000239, EGAD50000000240). All the code necessary to reproduce the figures of the paper is publicly available at https://github.com/bbglab/second-tumors-children. Further methodological descriptions can be found in the Supplementary Methods.

## Statement of significance

Using whole-genome and error-correcting ultra-deep duplex sequencing, we uncover different origins for second neoplasms in four children. We also uncover the presence of platinum-related mutations across 10 normal tissues of exposed individuals, highlighting the impact that the use of cytotoxic therapies may have in cancer survivors.
