# Supplementary material for: Origins of second tumors in children and mutational footprint of chemotherapy in normal tissues
Source: Cancer Discov. Author manuscript; Available in PMC 2024 Jun 4. (PMC11145171; doi:10.1158/2159-8290.CD-23-1186)
Supplement: Figure S1 [file EMS194327-supplement-Figure_S1.pdf]

Supplementary Figure 1

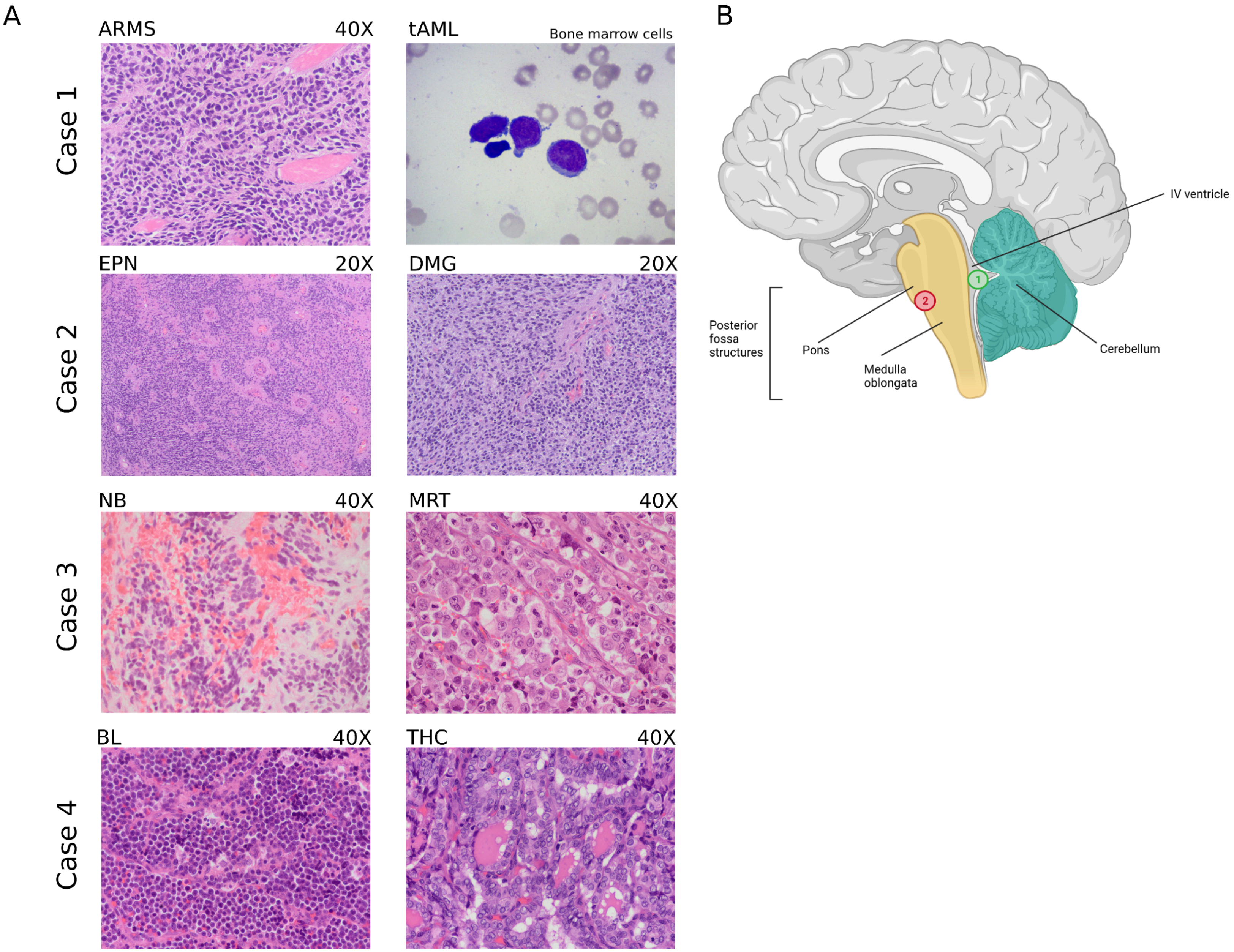

Supplementary Figure 1. Histopathology (H&E) analysis of tumor samples from cases 1, 2, 3 and 4.
A) Case 1 Alveolar Rhabdomyosarcoma (ARMS) and treatment-related Acute Myeloid Leukemia (tAML); Case 2 Ependymoma (EPN) and Diffuse Midline Glioma (DMG); Case 3 Neuroblastoma (NB) and Malignant Rhabdoid Tumor (MRT); Case 4 Burkitt Lymphoma (BL) and Thyroid Carcinoma (THC); B) Case 2 specific location in the brain of both the EPN (1, in the IVth ventricle) and DMG (2, in the right ponto-bulbar angle).
