# Supplementary material for: Origins of second tumors in children and mutational footprint of chemotherapy in normal tissues
Source: Cancer Discov. Author manuscript; Available in PMC 2024 Jun 4. (PMC11145171; doi:10.1158/2159-8290.CD-23-1186)
Supplement: Figure S2 [file EMS194327-supplement-Figure_S2.pdf]

Supplementary Figure 2

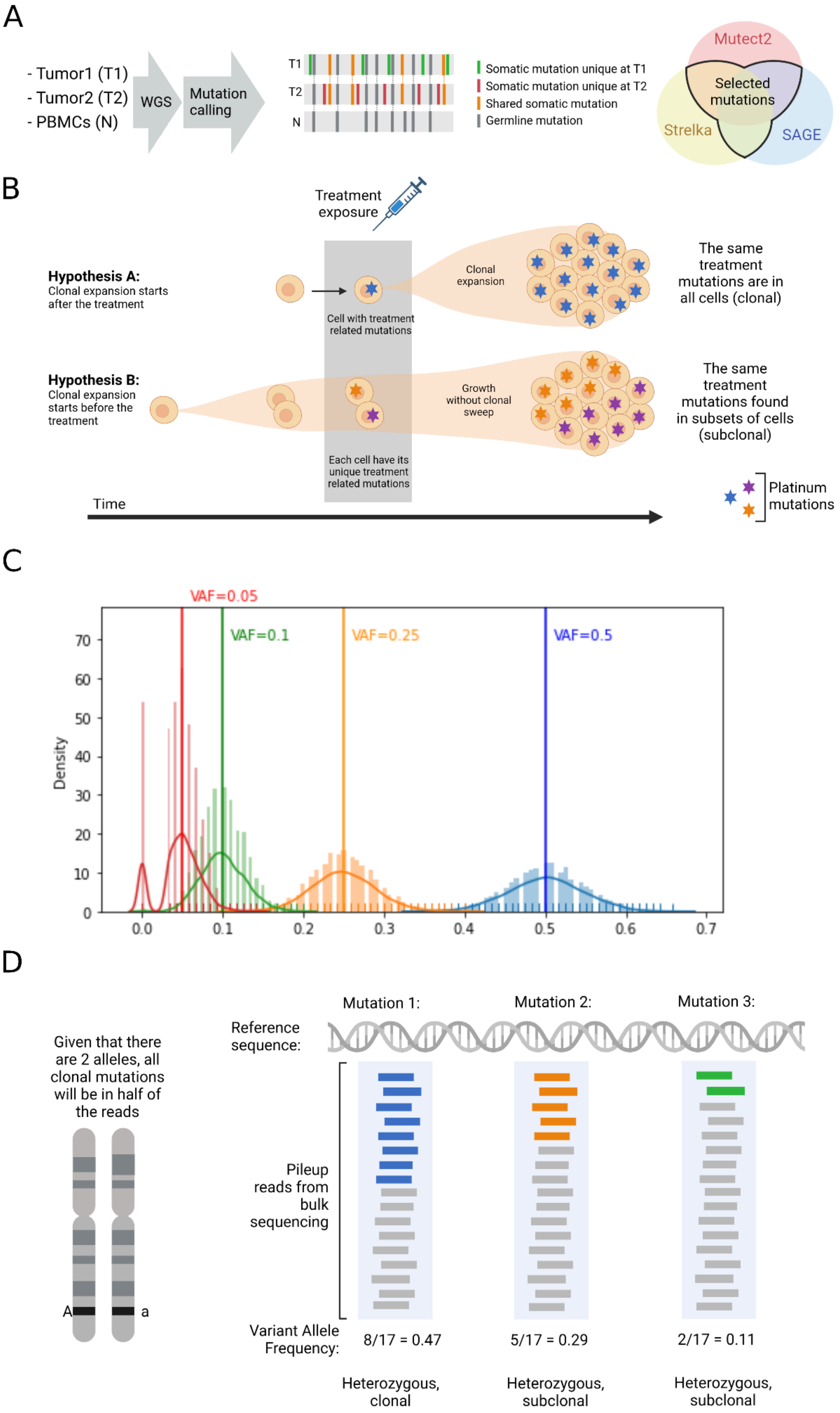

Supplementary Figure 2. Somatic mutation calling and timing of clonal expansion relative to the exposure to platinum.

A) Schematic diagram representing the analysis performed for all cases somatic calling using blood as germline reference. B) Toy representation of two cases of clonal expansion of a second malignancy upon exposure to platinum-based cytotoxic treatment. The top row represents an expansion from a single cell with platinum-related mutations. The second row presents a case where the second malignancy has expanded from two cells with platinum-related mutations. Color stars represent distinct sets of platinum-related mutations in different cells.

C) Distribution of variant allele frequency of platinum-related mutations in several cases of numbers of cells initiating the clonal expansion giving rise to the second tumor. (See text in Supplementary Information) VAF: Variant allele frequency.

D) Toy examples of the distribution of reads between the reference and the alternative alleles of three mutant sites. The distribution of the sequencing reads may be modeled following a Poisson process. The variant allele frequency of the variants is computed as the fraction between the reads mapping to the alternative allele and all the reads covering the position of the variant.
