# Supplementary material for: Origins of second tumors in children and mutational footprint of chemotherapy in normal tissues
Source: Cancer Discov. Author manuscript; Available in PMC 2024 Jun 4. (PMC11145171; doi:10.1158/2159-8290.CD-23-1186)
Supplement: Figure S3 [file EMS194327-supplement-Figure_S3.pdf]

Supplementary Figure 3

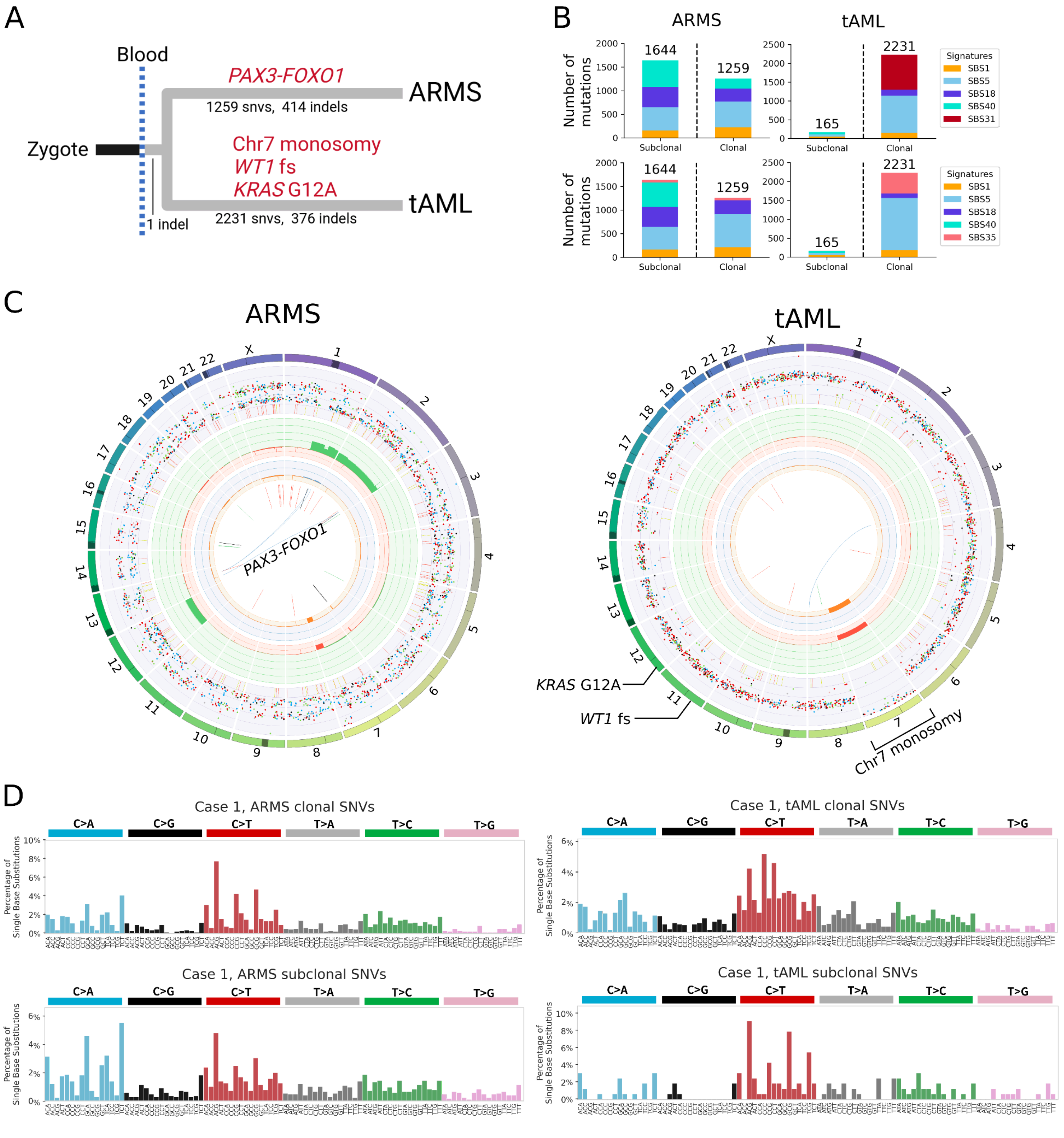

Supplementary Figure 3. Signature analysis and representation of somatic alterations of the two tumors of case 1.
A) Case 1 tree representing the total number of somatic SNVs and indels detected across the tumor samples. In Figure 1B the same tree, but only with the numbers of supporting SNVs is shown. This is because the calling of SNVs is less noisy than that of indels. B) Mutational signatures fitted on clonal and subclonal SNVs in both case 1 tumors. C) Circos plot representing all somatic alterations identified in tumors 1 and 2 of case 1. D) Mutational profile of clonal and subclonal mutations of both tumors.
