# Supplementary material for: Origins of second tumors in children and mutational footprint of chemotherapy in normal tissues
Source: Cancer Discov. Author manuscript; Available in PMC 2024 Jun 4. (PMC11145171; doi:10.1158/2159-8290.CD-23-1186)
Supplement: Figure S5 [file EMS194327-supplement-Figure_S5.pdf]

Supplementary Figure 5

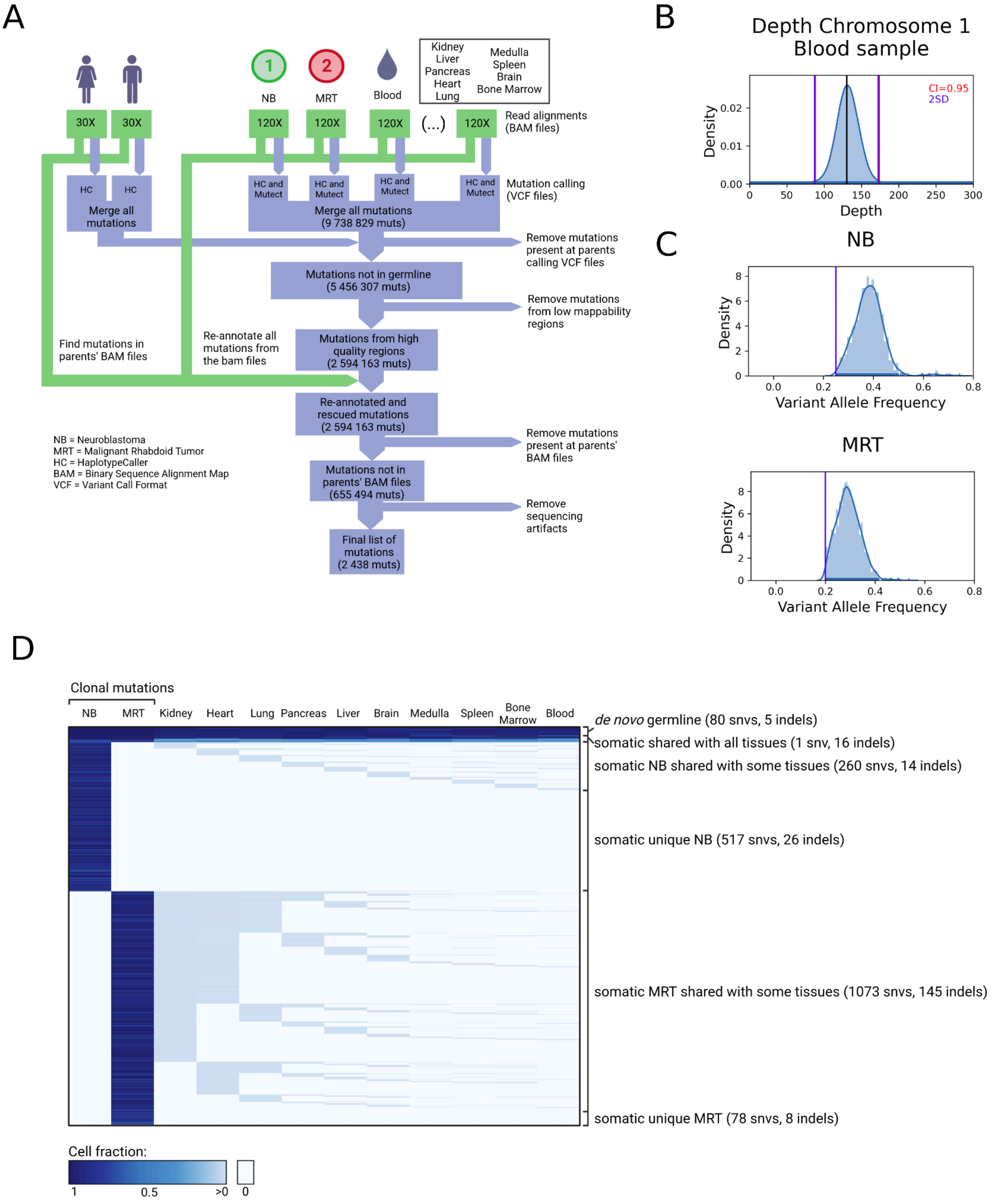

Supplementary Figure 5. Identification of somatic mutations shared between the two tumors and 10 normal tissues of case 3.

A) Schematic diagram representing all filtering steps applied to variants identified across the two tumors and 10 normal tissues of case 3. A detailed description of the process is provided in Methods of the Supplementary Material. B) Decision of lowest and highest depth considered as first filter of variants called within problematic mapping regions. The decision is exemplified with all mappable nucleotides of chromosome 1 in the blood sample. C) Selection of clonal mutations in both tumors to identify those shared across normal tissues. The threshold of VAF was set at 0.25 for the NB and at 0.2 for the MRT. D) Heatmap representing all somatic mutations (SNVs and Indels) and their cell fraction shared with 10 normal tissues. Of all clonal mutations in the NB, 274 are shared with at least one normal tissue –resulting most likely from their shared developmental origin–, while 543 are unique to the NB, probably having accumulated since its most recent common ancestor cell separated from the lineages that gave rise to all normal tissues represented. The proportions of shared and unique clonal mutations of the MRT are very different (1218 and 86, respectively). Some of the 1218 shared mutations probably result too from the shared developmental origin of the MRT and the 10 normal tissues. However, the sheer quantity of shared mutations suggests the presence of metastatic MRT cells within some or all the 10 normal tissues sequenced, which would have contributed the majority of these common mutations. Given that both shared developmental mutations and mutations resulting from metastatic infiltration are present at roughly the same VAF, it is virtually impossible to separate them in this case.
