# Supplementary material for: Origins of second tumors in children and mutational footprint of chemotherapy in normal tissues
Source: Cancer Discov. Author manuscript; Available in PMC 2024 Jun 4. (PMC11145171; doi:10.1158/2159-8290.CD-23-1186)
Supplement: Figure S6 [file EMS194327-supplement-Figure_S6.pdf]

Supplementary Figure 6

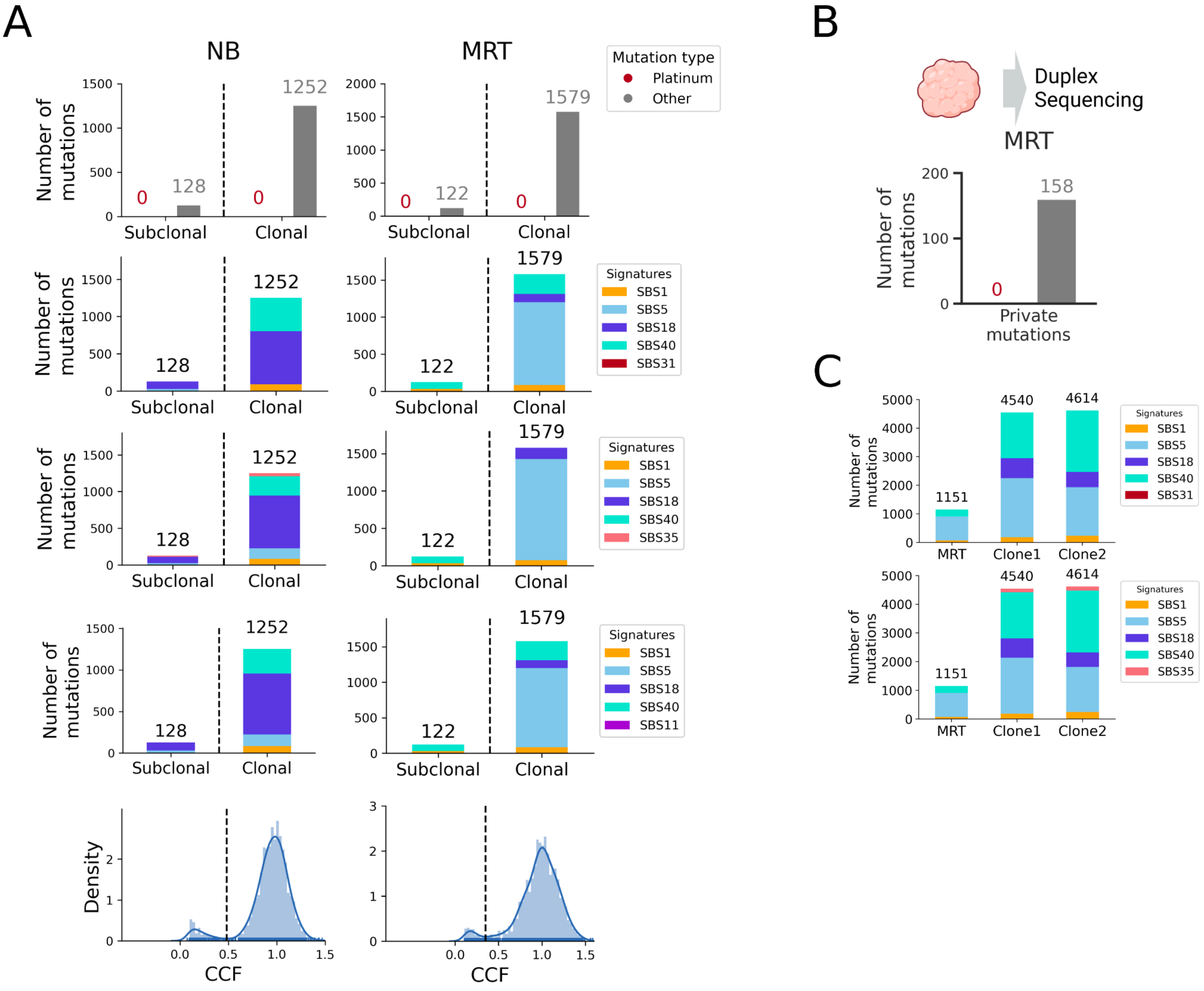

Supplementary Figure 6. Signature analysis of case 3.

A) Mutational signatures fitted on clonal and subclonal SNVs in the two tumors. The uppermost panel of the figure is analogous to main Figure 1D and 2C (case 1 and 2, respectively), while the bottom panels present the results of the test for the presence of platinum-related mutations (SBS31 and SBS35) and temozolomide-related mutations (SBS11). No trace of the platinum or the temozolomide mutational signatures was found within the mutations of either tumor. Note that the number of clonal SNVs is slightly lower than in Figure 3B; this is because it has been calculated from the somatic mutation calling using only the blood sample as the normal-matched tissue (see Supplementary methods*)*. B) Mutational signatures fitted on the private SNVs of the MRT sample identified through the error corrected duplex bulk DNA sequencing. C) Mutational signatures fitted on clonal SNVs of the MRT sample and the two cell lines derived from MRT single cells.
