# Supplementary material for: Origins of second tumors in children and mutational footprint of chemotherapy in normal tissues
Source: Cancer Discov. Author manuscript; Available in PMC 2024 Jun 4. (PMC11145171; doi:10.1158/2159-8290.CD-23-1186)
Supplement: Figure S7 [file EMS194327-supplement-Figure_S7.pdf]

Supplementary Figure 7

A

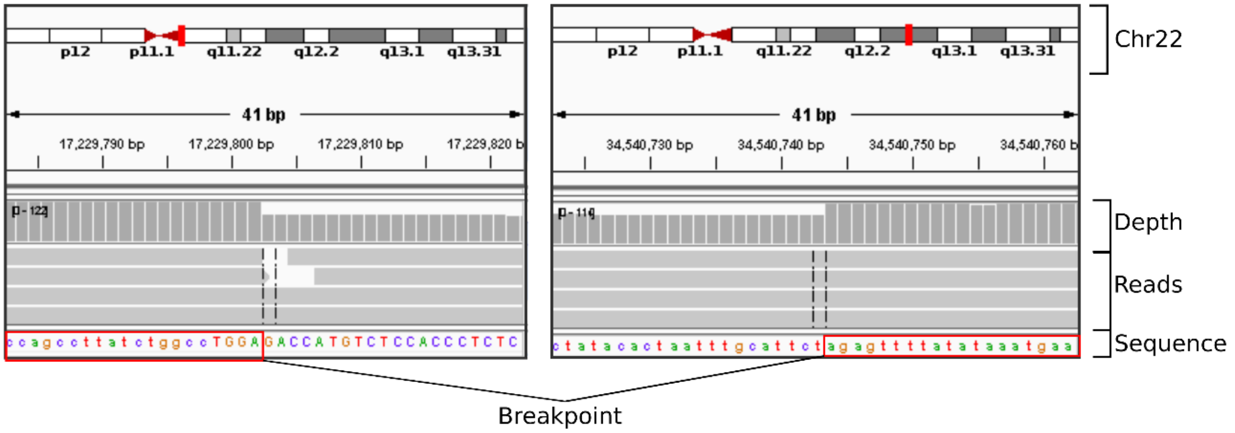

B

	SMARCB1 large deletion			SMARCB1 small indel		
	Mutation	WT	Depth	Mutation	WT	Depth
Rhabdoid tumor	34	56	90	31	43	74
Heart	3	80	83	2	85	87
Kidney	0	81	81	0	81	81
Liver	1	90	91	0	91	91
Pancreas	1	92	93	0	76	76
Blood	0	88	88	0	90	90
Neuroblastoma	0	83	83	0	70	70

Sequence Mutation	CTGGCCTGGAAGAGTTTTAT	GAACATCCATAAACATTTCC
Sequence WT	CTGGCCTGGAGACCATGTCT	GAACATCCATGTGGGAAACATTTCC

C

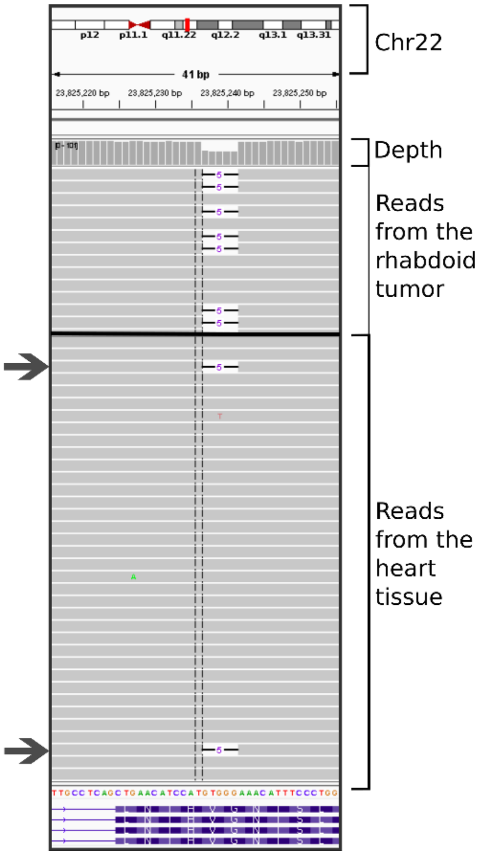

D

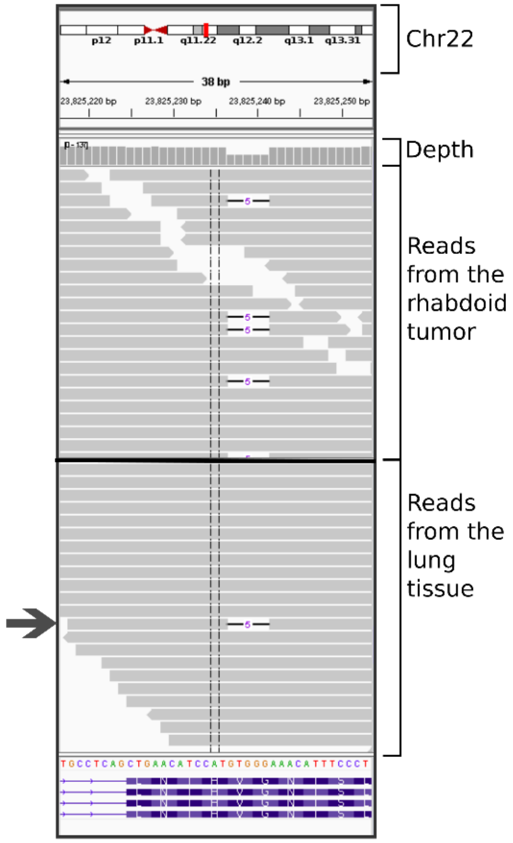

Supplementary Figure 7. Visualization of WGS alignment data showing evidence of *SMARCB1* driver alterations in normal tissues from case 3.

A) Left and right flanks of the breakpoint of the large deletion in chromosome 22. B) Table summarizing the number of reads WT and with the *SMARCB1* alterations. C) Visualization of the 2 *SMARCB1* indel variants found in heart sample. D) Visualization of the *SMARCB1*-indel read in lung tissue. All snapshots correspond to sequencing read alignments visualized using the IGV.
