# Supplementary material for: Origins of second tumors in children and mutational footprint of chemotherapy in normal tissues
Source: Cancer Discov. Author manuscript; Available in PMC 2024 Jun 4. (PMC11145171; doi:10.1158/2159-8290.CD-23-1186)
Supplement: Figure S8 [file EMS194327-supplement-Figure_S8.pdf]

Supplementary Figure 8

A

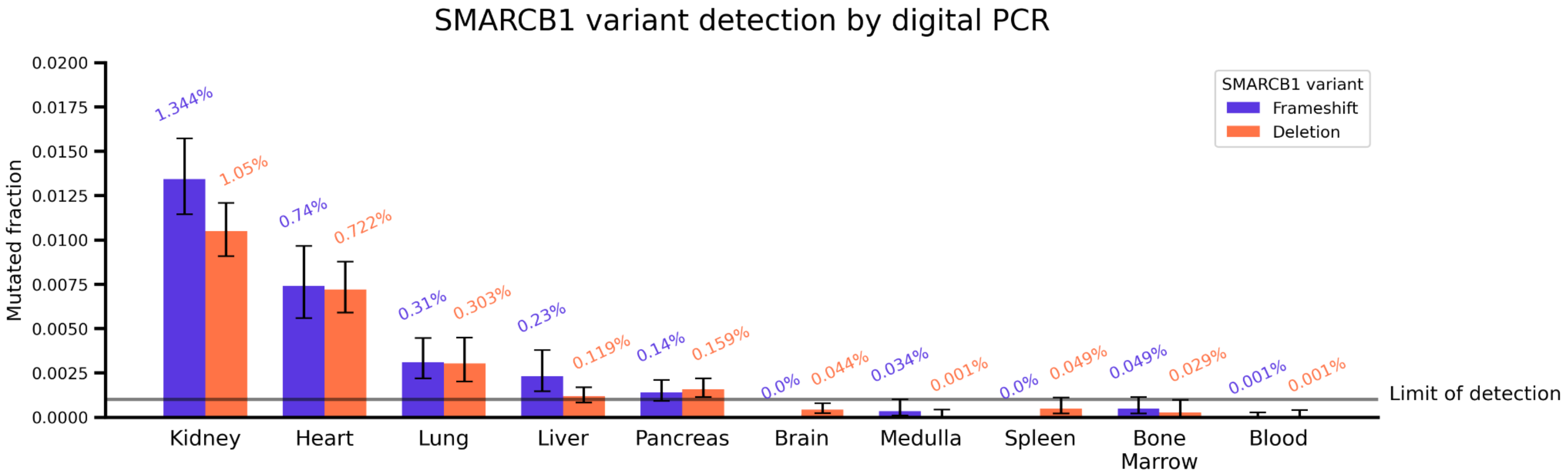

B

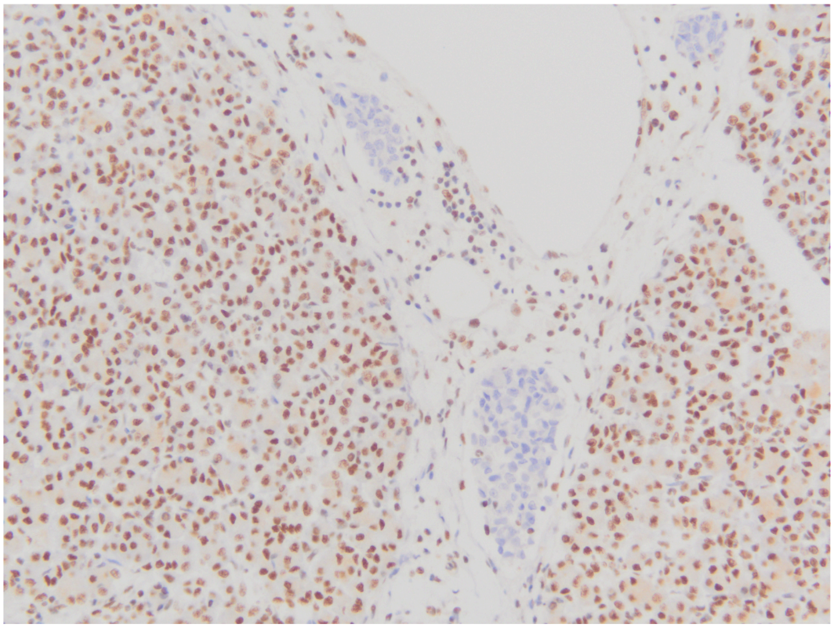

INI1 IHC
Pancreas

Supplementary Figure 8. *SMARCB1* null cells identified in normal tissues of case 3.
A) Results of digital PCR for the two *SMARCB1* mutations across 10 normal tissues of case 3. B) Immunohistochemistry (IHC) INI1 protein in normal pancreas tissue. This demonstrates the presence of MRT cells infiltrating the normal tissue.
