# Supplementary material for: Origins of second tumors in children and mutational footprint of chemotherapy in normal tissues
Source: Cancer Discov. Author manuscript; Available in PMC 2024 Jun 4. (PMC11145171; doi:10.1158/2159-8290.CD-23-1186)
Supplement: Figure S9 [file EMS194327-supplement-Figure_S9.pdf]

Supplementary Figure 9

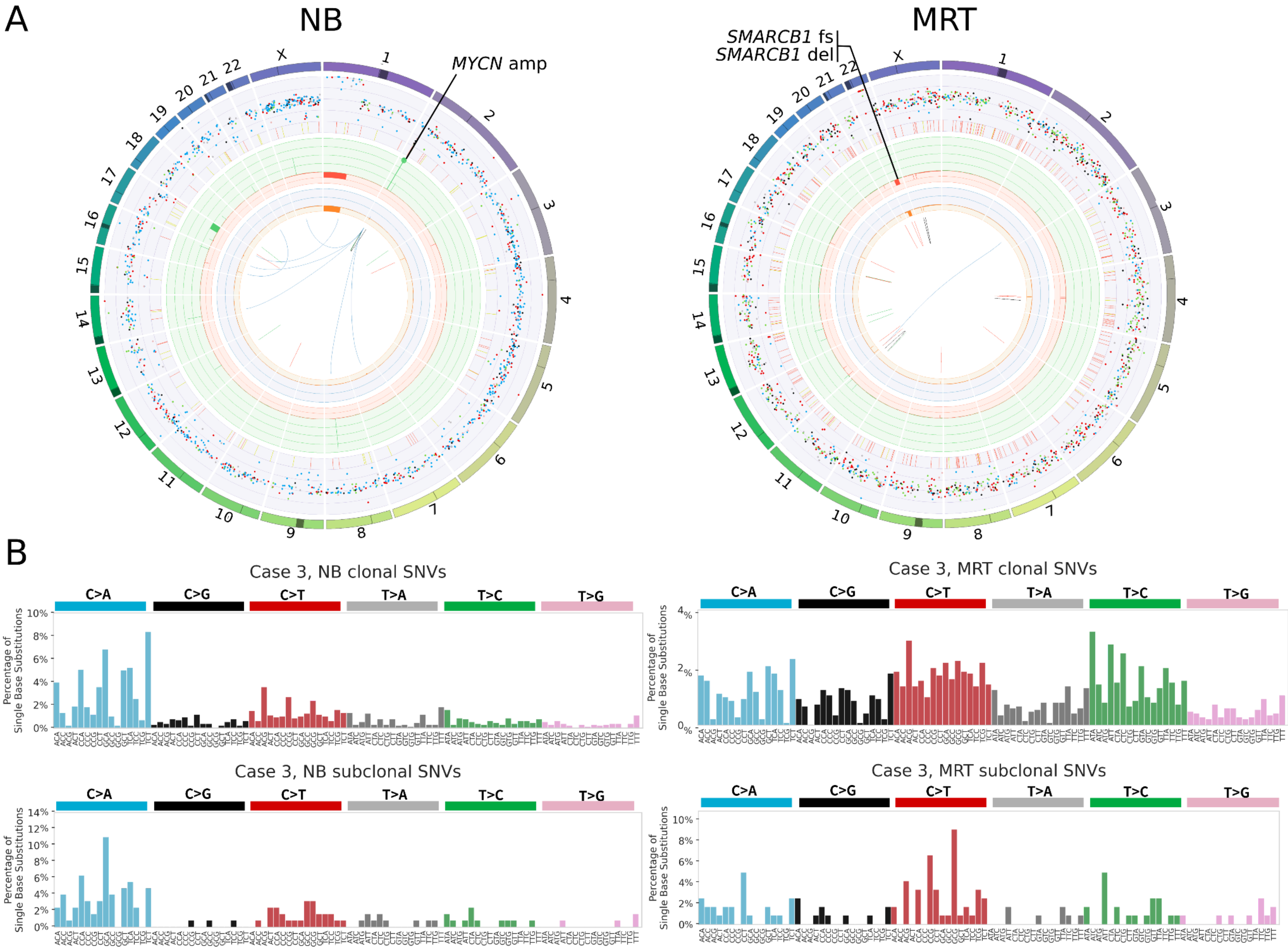

Supplementary Figure 9. Representation of somatic alterations of the two tumors of case 3.
A) Circos plot representing all somatic alterations identified in tumors 1 and 2 of case 3.
B) Mutational profile of clonal and subclonal mutations of both tumors.
