# Supplementary material for: Origins of second tumors in children and mutational footprint of chemotherapy in normal tissues
Source: Cancer Discov. Author manuscript; Available in PMC 2024 Jun 4. (PMC11145171; doi:10.1158/2159-8290.CD-23-1186)
Supplement: Figure S10 [file EMS194327-supplement-Figure_S10.pdf]

Supplementary Figure 10

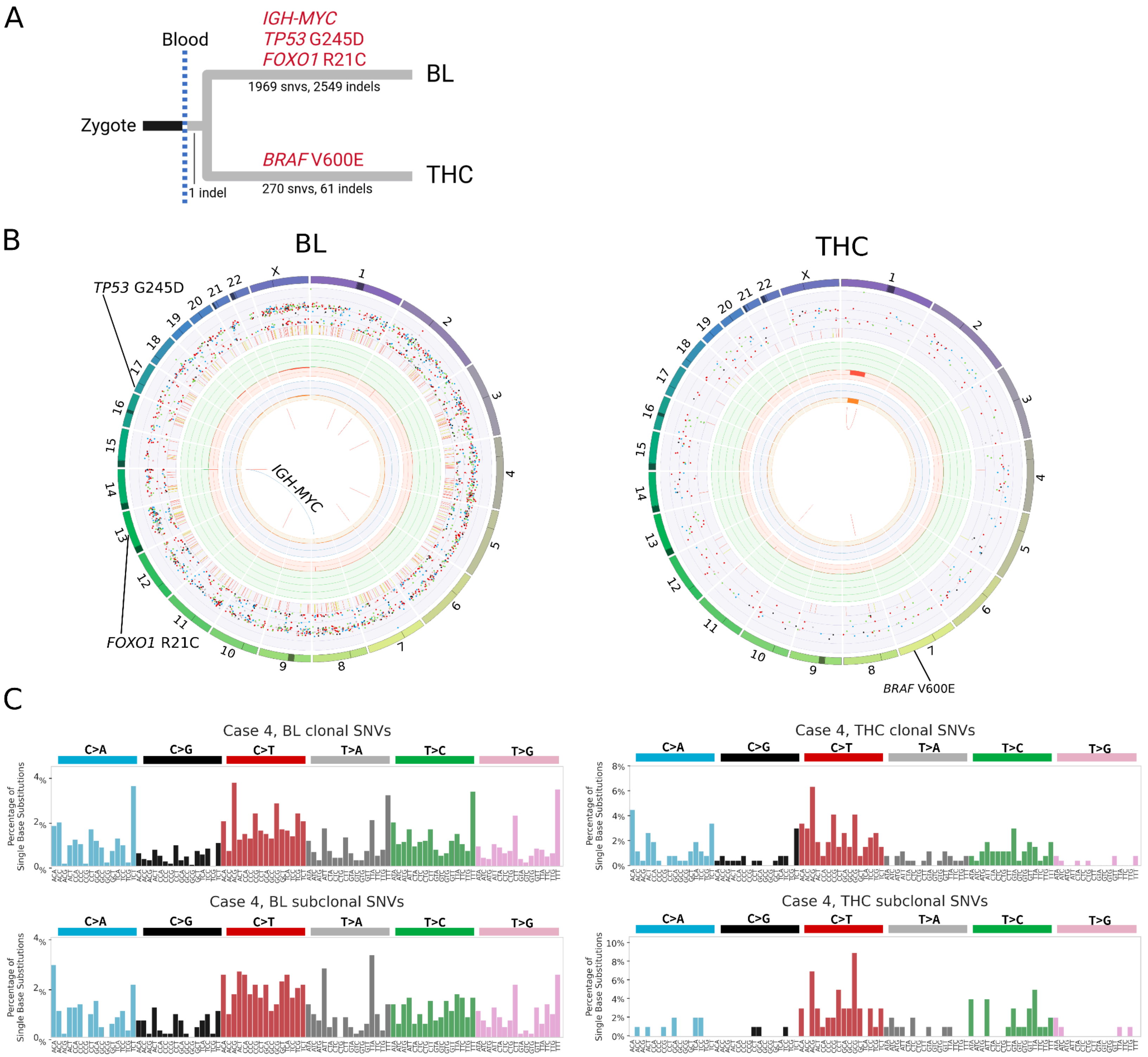

Supplementary Figure 10. Representation of somatic alterations of the two tumors of case 4.
A) Tree representing the total number of somatic SNVs and indels detected across the tumor samples.
B) Circos plot representing all somatic alterations identified in tumors 1 and 2 of case 4.
C) Mutational profile of clonal and subclonal mutations of both tumors.
