# Supplementary material for: Origins of second tumors in children and mutational footprint of chemotherapy in normal tissues
Source: Cancer Discov. Author manuscript; Available in PMC 2024 Jun 4. (PMC11145171; doi:10.1158/2159-8290.CD-23-1186)
Supplement: Figure S11 [file EMS194327-supplement-Figure_S11.pdf]

Supplementary Figure 11

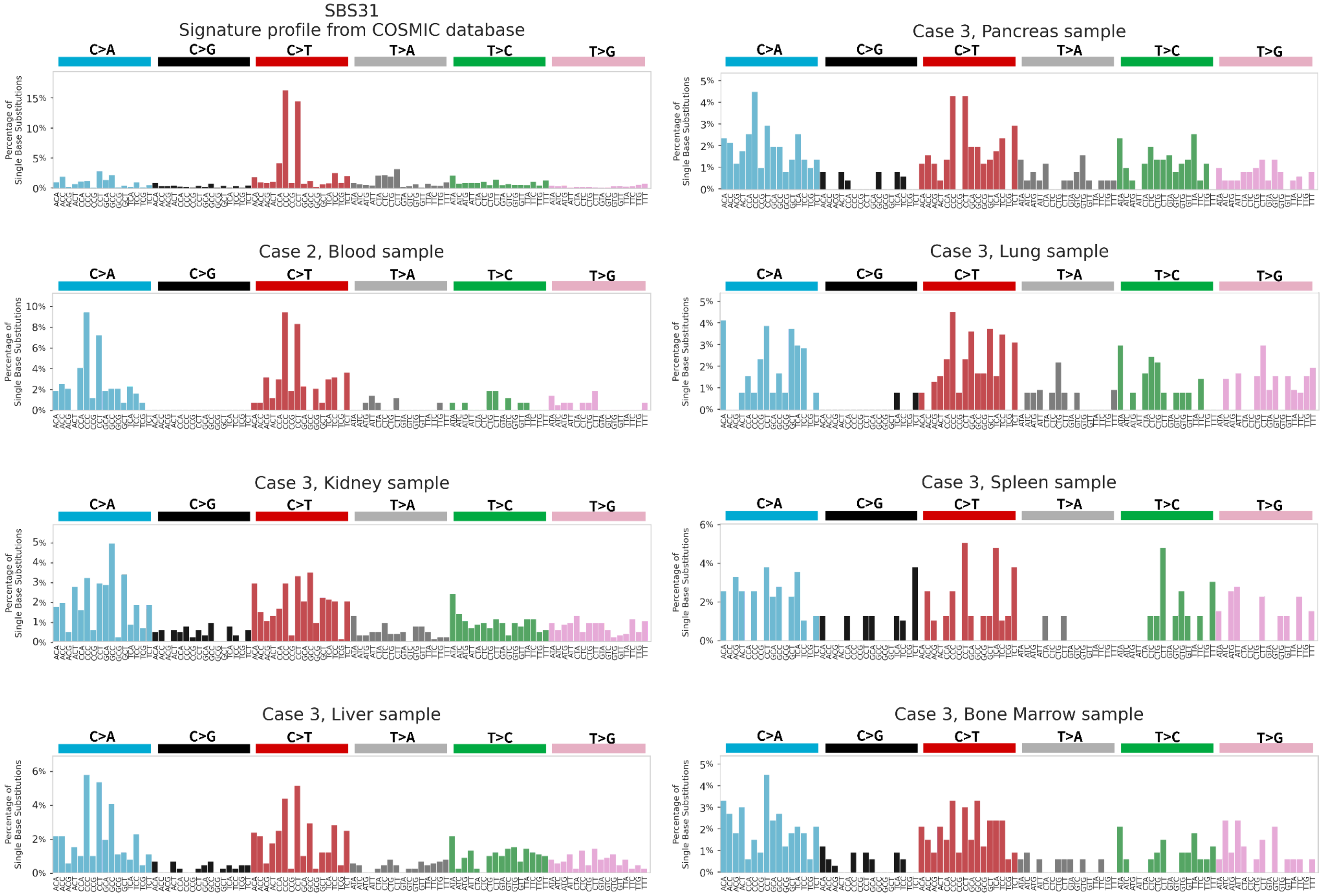

Supplementary Figure 11. Mutational profiles of the samples sequenced with Duplex Sequencing. Upper-left panel is the SBS31 mutational profile from the COSMIC database. Depicted are the profiles from case 2 blood sample and case 3 kidney, liver, pancreas, lung, spleen and bone marrow samples.
