# Supplementary material for: Origins of second tumors in children and mutational footprint of chemotherapy in normal tissues
Source: Cancer Discov. Author manuscript; Available in PMC 2024 Jun 4. (PMC11145171; doi:10.1158/2159-8290.CD-23-1186)
Supplement: Figure S12 [file EMS194327-supplement-Figure_S12.pdf]

Supplementary Figure 12

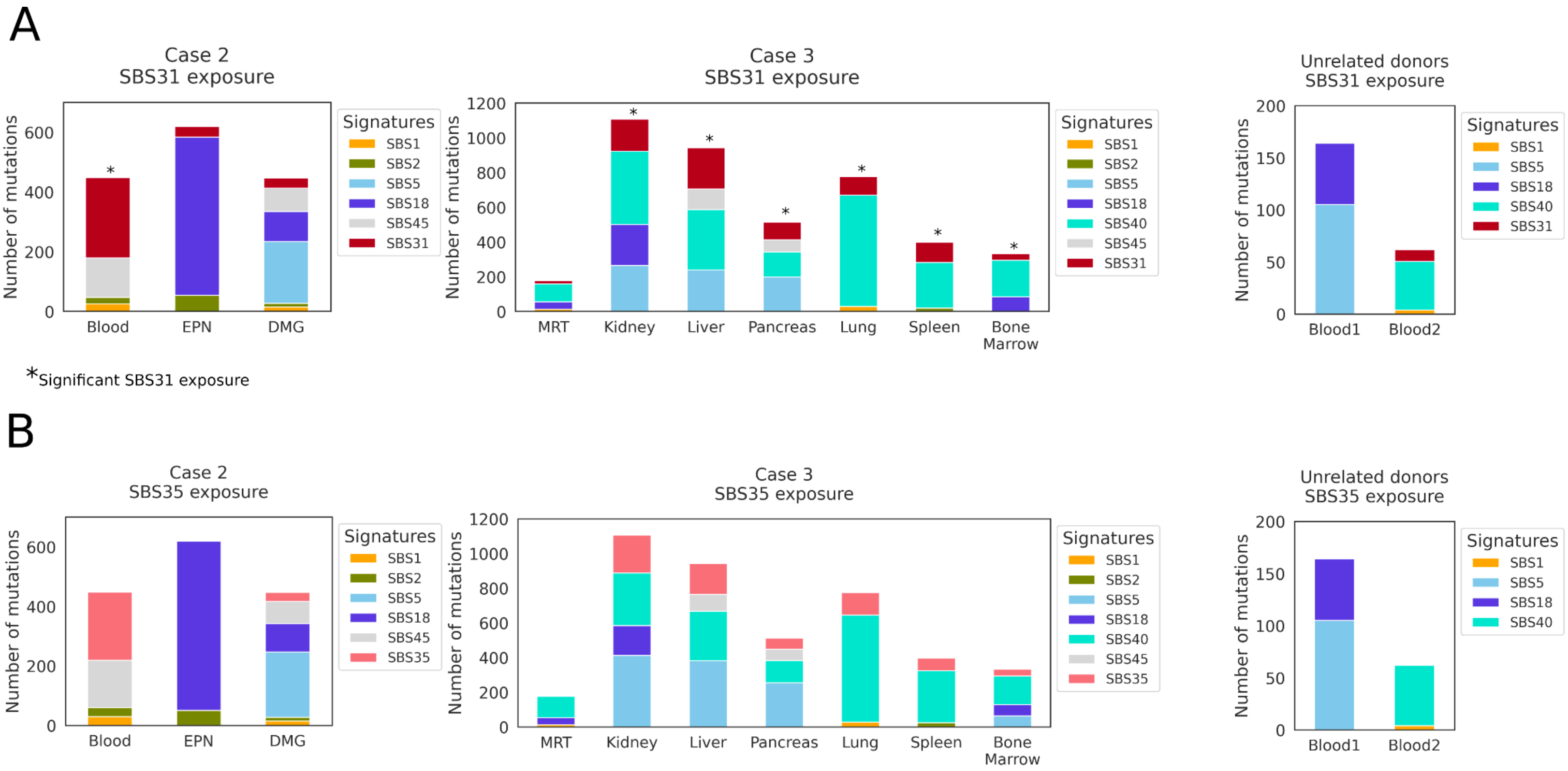

Supplementary Figure 12. Signature fitting analysis to assess the activity of SBS31 and SBS35 across samples.
A) SBS31 and B) SBS35 fitting analysis of blood, EPN and DMG samples from case 2 (left panel); MRT, kidney, liver, pancreas, lung, spleen and bone marrow samples from case 3 (middle panel); and blood samples from unrelated untreated donors (right panel). Asterisks denote specific SBS31 exposure by the specificity analysis performed (see *Platinum signatures specificity analysis* in Supplementary Methods).
