# Supplementary material for: Origins of second tumors in children and mutational footprint of chemotherapy in normal tissues
Source: Cancer Discov. Author manuscript; Available in PMC 2024 Jun 4. (PMC11145171; doi:10.1158/2159-8290.CD-23-1186)
Supplement: Figure S13 [file EMS194327-supplement-Figure_S13.pdf]

Supplementary Figure 13

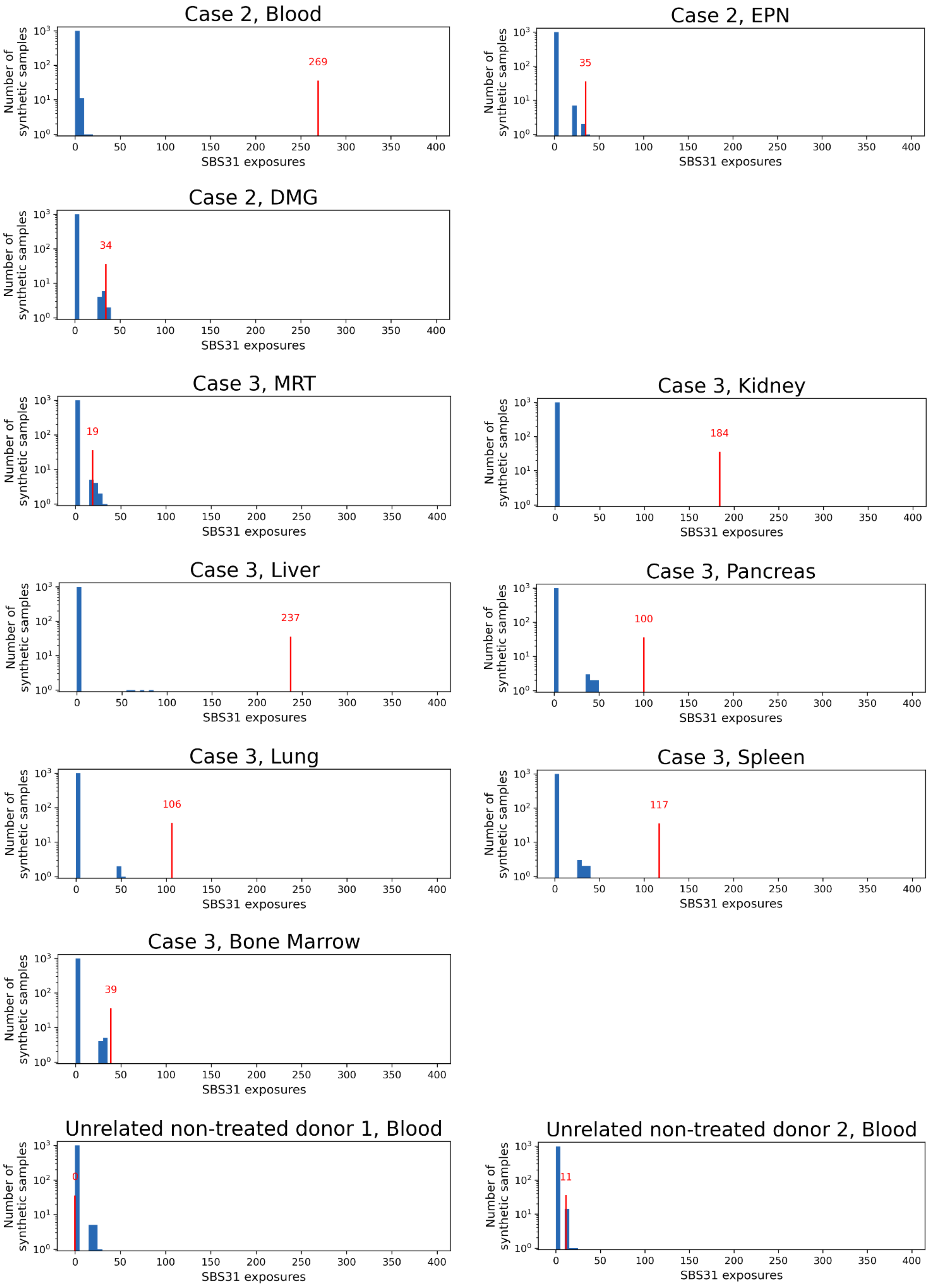

Supplementary Figure 13. mSigAct SBS31 detection specificity test.

One thousand synthetic samples constructed with the contributions of different signatures (excluding SBS31/35) fitted for every normal tissue (or tumor) using the mSigAct tool (see Methods) were run through mSigAct testing for the presence of SBS31. In all cases, except the MRT, EPN, DMG and unrelated donor 2 blood samples, the exposure of SBS31 observed in the real sample was outside the boundaries of the distribution of the exposure of the synthetic samples.

Case 2 blood and DMG samples; case 3 MRT, kidney, liver, pancreas, lung, spleen and bone marrow samples; Unrelated non-treated donor 1 and 2 blood samples.
