# Supplementary material for: Origins of second tumors in children and mutational footprint of chemotherapy in normal tissues
Source: Cancer Discov. Author manuscript; Available in PMC 2024 Jun 4. (PMC11145171; doi:10.1158/2159-8290.CD-23-1186)
Supplement: Figure S14 [file EMS194327-supplement-Figure_S14.pdf]

Supplementary Figure 14

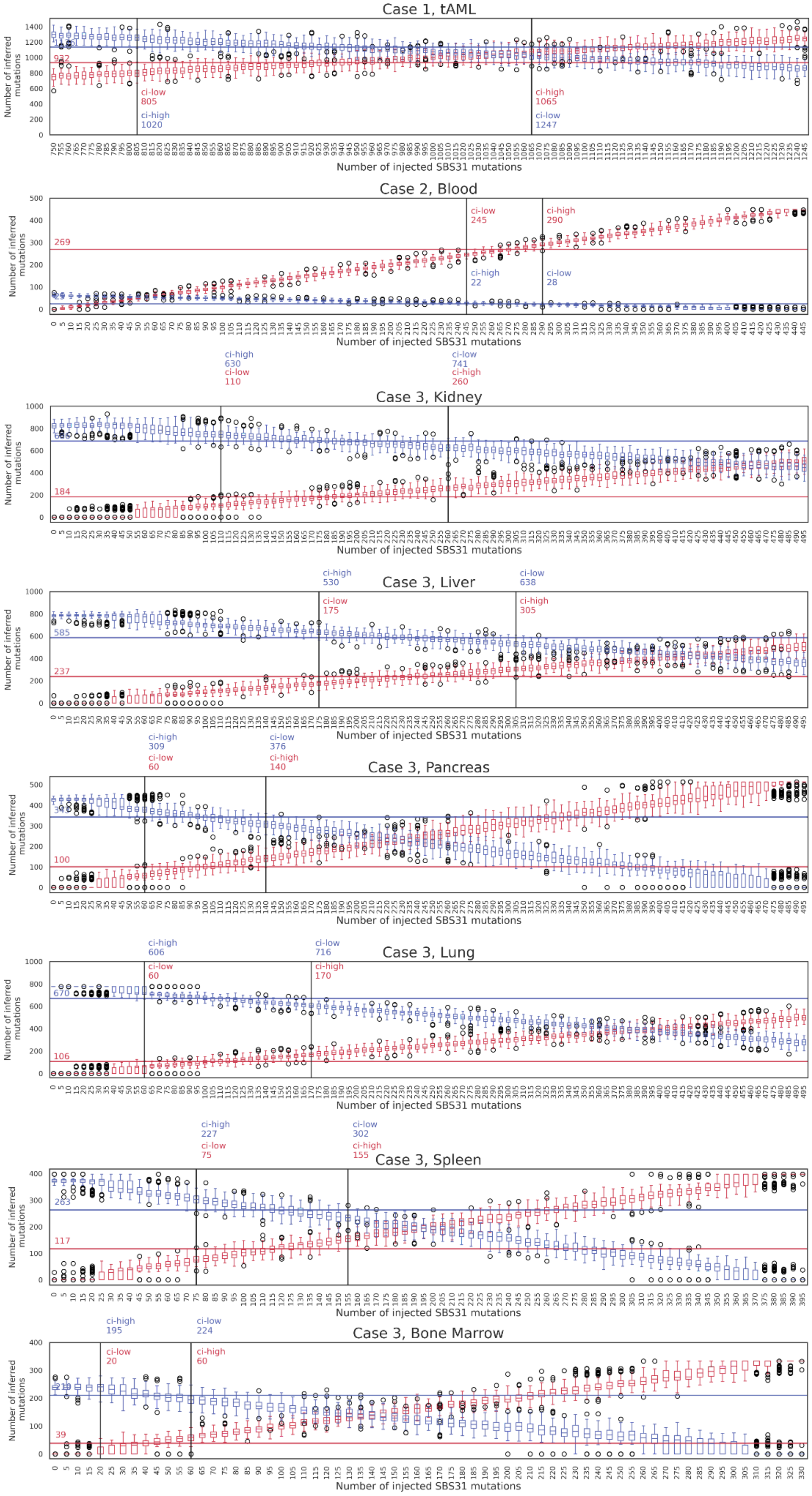

Supplementary Figure 14. Calculation of confidence intervals of activity of SBS31.

We built one synthetic sample with the contributions of different signatures (excluding SBS31/35) fitted for every normal tissue (or tumor). Then, an increasing number of platinum mutations (i.e., following the tri-nucleotide probabilities of SBS31) were injected into these samples, and after every injection, the exposure to SBS31 and aging mutational processes (SBS1+SBS5+SBS40) were computed 100 times using mSigAct. Then, by intersecting the obtained distributions with horizontal lines set at the positions corresponding to the observed exposure of platinum and age-related mutations provided confidence intervals to these values. (See details in Methods.)

Case 1 tAML; Case 2 blood sample; Case 3 kidney, liver, pancreas, lung, spleen and bone marrow samples; Unrelated non-treated donor 1 and 2 blood samples.
