# Supplementary material for: Origins of second tumors in children and mutational footprint of chemotherapy in normal tissues
Source: Cancer Discov. Author manuscript; Available in PMC 2024 Jun 4. (PMC11145171; doi:10.1158/2159-8290.CD-23-1186)
Supplement: Supplementary information [file EMS194327-supplement-Supplementary_information.pdf]

Supplementary Information

Origin of second tumors in four children and the mutational footprint of chemotherapy in normal tissues

Mònica Sánchez-Guixé[1,2], Ferran Muiños[1], Morena Pinheiro-Santin[1], Víctor González-Huici[1], Carlos J. Rodriguez-Hernandez[3], Alexandra Avgustinova[3], Cinzia Lavarino[4], Abel González-Pérez[1,2,*], Jaume Mora[4], Núria López-Bigas[1,2,5,*]

Affiliations

1. Institute for Research in Biomedicine (IRB Barcelona), The Barcelona Institute of Science and Technology, Baldiri Reixac, 10, 08028 Barcelona, Spain.
2. Centro de Investigación Biomédica en Red en Cáncer (CIBERONC), Instituto de Salud Carlos III, Madrid, Spain.
3. Institut de Recerca Sant Joan de Déu, Barcelona 08950, Spain.
4. Pediatric Cancer Center Barcelona (PCCB). Hospital Sant Joan de Déu. 08950 Barcelona. Spain
5. Institució Catalana de Recerca i Estudis Avançats (ICREA), Barcelona, Spain

* AG-P and NL-B contributed equally to this work

Correspondence should be addressed to jaume.mora@sjd.es and nuria.lopez@irbbarcelona.org

Table of Contents

Supplementary Methods..3
Genomic analysis with normal-matched samples...3
Mutational signature analysis on tumor clonal and subclonal SNVs...................................... 3
Analysis of germline variants..3
Identification of somatic mutations across tumors and normal tissues (case 3).....................4
Detection of SMARCB1 mutations in MRT and normal tissues (case 3)................................5
Duplex sequencing mutation calling.. 5
Mutational signature analysis of normal tissues.. 5
Mutational signature analysis in MRT-derived cell lines.. 6
Mutational signature refitting..6
Platinum signatures specificity analysis..7
Inferred activity of platinum-related signatures across tumors and normal tissues................ 8
Somatic SNVs accumulated by the tumors in their process of clonal expansion................... 9
Confidence intervals of inferred exposure of platinum-related signature................................9
SNV rate per day.. 10
Timing the clonal expansion of a second tumor based on platinum mutations.....................10
Probability of KRAS mutation (tAML) generated by the platinum signature......................... 11
Full clinical information of the four children..12
References.. 16

Supplementary Methods

Genomic analysis with normal-matched samples

We identified somatic mutations (SNVs and indels) in the eight tumors through direct comparison with their matched normal blood samples. The sequencing reads were first aligned to the GRCh38 human reference genome using BWA version 0.7.178. Then, variants called by two or more of Mutect2(1), Strelka2(2) and SAGE (https://github.com/hartwigmedical/hmftools/tree/master/sage) were identified as true somatic mutations (Supplementary Fig. S2A). Mutations present at gnomAD(3) variants database at a frequency higher than 0.0001 were discarded. The HaplotypeCaller from GATK (v4.3.0.011)(4) was used to identify germline variants from the sequences of the normal blood samples of the four cases.

The Cancer Cell Fraction (CCF) of mutations was calculated using the following formula:

$$CCF = \frac{VAF \times (Purity \times CN + (1 - Purity) \times 2)}{Purity}$$

where the Variant Allele Frequency (VAF) is calculated dividing the reads supporting the alternate allele by all reads covering the genomic position of the mutation. The Copy Number (CN) at each genomic position and the Purity are calculated via the Purple tool developed by the Hartwig Medical Foundation(5,6) pipeline (https://github.com/hartwigmedical/hmftools/tree/master/sage). To distinguish clonal from subclonal mutations, we set up arbitrary thresholds at the value of CCF that visually offered the best separation between the two sets (Fig. 1D and Supplementary Fig. S3B). In the figures of the main manuscript only numbers of SNVs are shown, as their identification via somatic calling methods is more accurate. The number of mutations (SNVs and indels) identified in the tumors of cases 1, 2, and 4, shown in Supplementary Figures S3A, S4A, and S10A were used in the reconstruction of their evolutionary histories. The somatic mutations of the tumors of case 3 were called through a more elaborate comparison taking into account the variants observed across seven normal tissues and the parents' normal blood (see below).

Mutational signature analysis on tumor clonal and subclonal SNVs

Mutational processes were analyzed using the mutational signature fitting tool mSigAct(7) for the clonal and subclonal mutations of each tumor. We tested whether either of the platinum-related mutational signatures SBS31 and SBS35 improved the fitting of the mutational profile, and calculated the number of mutations assigned to these signatures respectively. The methodology to study mutational signatures in normal tissues is described below.

Analysis of germline variants

Germline variants identified through HaplotypeCaller were processed with VEP(8) and population allele frequencies of the variants were annotated with gnomAD v3.0.0 database(3) For each child, we annotated variants with a gnomAD allele frequency below 0.01, which potentially affected the protein sequence (frameshift, nonsense, missense, inframe, splicing variant) of any within a list of genes associated with cancer predisposition. The list (216 genes) was curated as the union of three lists put together by Rahman et al Nature 2014(9) (114 genes), Fiala et al Nature 2021(10) (90 genes), and Akhavanfard et al NatCom 2020(11) (204 genes). The resulting lists of germline variants affecting these genes for all children appear in Table 1. In all cases, these variants appeared in heterozygosis and in neither of the cases there was a second somatic mutation (which would affect the other allele) observed. An analysis of

structural variants with GRIDSS(12,13) showed no deleterious germline or somatic alteration affecting any of the genes in the susceptibility list. In conclusion, no clear germline predisposition variant underlying the presence of the two tumors was found in any of the children.

Identification of somatic mutations across tumors and normal tissues (case 3)

In addition to the regular somatic mutation calling carried out on the tumor samples of all individuals (see above), we decided to perform a more thorough ad-hoc process to identify the somatic mutations in the tumors of case 3 using as reference not only the blood sample, but the samples from normal tissues taken during diagnosis or at autopsy. This process began with all variants identified by the HaplotypeCaller from GATK version 4.3.0.0(4) across the two tumor samples and the samples from 10 normal tissues (see above). We also identified potential low-VAF somatic mutations using Mutect2(14) run in single-sample mode on each of the 12 samples. In each sample, mutations detected by either Mutect2 or HaplotypeCaller were considered. In the blood samples from both parents, we identified variants using HaplotypeCaller.

Variants identified in each sample were merged into a table (with variants as rows and tissues as columns) and several filters were subsequently applied (Supplementary Fig. S5A). First, variants that were in common with any parent (represented by any number of reads, down to only one) were considered germline and thus removed. Then, we removed variants called in regions with unusually low or high coverage (i.e., outside 95% of the coverage distribution of each chromosome in each sample) (Supplementary Fig. S5B). Variants detected in any tissue were manually searched for in the BAM files of all other tissues and “rescued” if they were found to be supported (even by a single read). Finally, we applied a filter to remove possible artifacts from the sequencer by annotating recurrent altered positions across blood samples from 25 unrelated children. To do this, we first discarded all mutations present in all blood samples from the cohort. Second, we applied a beta-binomial test(15) using the distribution of all nucleotides at each genomic position across the blood samples from the 25 unrelated children. We removed variants whose distribution of alternates fell within the beta-binomial distribution (FDR corrected q-value $< 10^{-5}$). Third, for variants not present across the blood samples of these 25 children, we discarded all variants that did not appear as clonal in any of the two tumors (VAF>0.25 NB or VAF>0.2 in MRT; Supplementary Fig. S5C), variants present in only one normal tissue, and variants supported by less than 15 reads in the sample with the highest support.

Then, to identify *de novo* germline mutations among the remaining variants we carried out a one-sided binomial test to evaluate if their observed VAF was 0.5 or higher, considering an FDR corrected q-value threshold of 10^{-5} as explained in reference No.(15). All the variants (N=68) for which we could not reject the null hypothesis (VAF>=0.5) at q-value=10^{-5} across all tissues were considered *de novo* germline mutations. All other variants (appearing in at least two tissues) were considered somatic mutations, acquired throughout development.

The results of this analysis, detailing the number of clonal somatic mutations (SNVs and indels) of the NB and the MRT shared across 10 normal tissues of case 3 are represented in Supplementary Fig. S5D. The somatic mutations (SNVs and indels) identified in the NB and the MRT samples were used to track the evolutionary history of both tumors of case 3 in our study (Fig. 2B and Supplementary Fig. S5D).

Detection of *SMARCB1* mutations in MRT and normal tissues (case 3)

We observed that a higher fraction of clonal mutations of the MRT were shared across normal tissues than of clonal mutations of the NB (Supplementary Fig. S5D). This prompted us to speculate about the possibility that infiltrating MRT cells were present in these normal tissues. To decipher whether the *SMARCB1* driving events were detected in these normal tissues, we first carried out a manual inspection using *samtools view*(16,17) in the corresponding BAM files for the *SMARCB1* mutations detected in the MRT. Specifically, we searched for a chimeric 20 nucleotide-long sequence formed by 10 nucleotides at each side of the breakpoints forming the ~17 MB-long chr22 deletion of the *SMARCB1* gene (Supplementary Fig. S7A) and a chimeric *SMARCB1* sequence subtracting the 5 nucleotides deleted by the small indel/frameshift in the other hit of the gene (Supplementary Fig. S7B). We found 3 reads in the heart sample, 1 read in the liver sample and 1 read in the pancreas sample that contained the 20 nucleotide-long chimeric sequence and 2 reads in the heart sample and 1 read in the lung sample that contained the small indel/frameshift chimeric sequence. Supplementary Figures S7C and D illustrate the reads containing the small indel/frameshift chimeric sequence in the heart and lung samples, respectively, presented as snapshots from the Integrative Genomics Viewer (IGV)(18).

To probe the extent of the presence of the *SMARCB1* variants across all 10 normal tissues, we next designed two digital PCR (Supplementary Fig. S8A) probes targeting the two chimeric sequences. The results showed that both *SMARCB1* alterations were present at low frequency in the kidney, heart, liver and pancreas normal samples. The mutated fraction of cells carrying the two alterations was similar in each tissue, suggesting the presence of *SMARCB1* null cells in these tissues. For other tissues, the values were below the limit of detection, so the presence of the alterations could not be probed.

These results suggested the presence of MRT cells across these normal tissues, which was confirmed by immunohistochemistry analysis of the INI1 protein , the product of the *SMARCB1* gene. This analysis revealed INI1 negative rhabdoid cells infiltrating the blood vessels of the pancreas (Supplementary Fig. S8B), confirming a metastatic spreading of the MRT in the normal tissues as the most likely explanation of the presence of *SMARCB1* alterations (and other clonal somatic mutations of the MRT) at a low cellular fraction across the normal tissues.

Duplex sequencing mutation calling

DNA sequencing of the Duplex libraries was carried out in a NovaSeq 6000 S4 platform. The alignment and mutation calling analysis were performed using the proprietary TwinStrand DuplexSeq Mutagenesis AppTM at DNAnexus. The mutagenesis panel was used in all samples (48Kbp of a genome representative panel targeting 20 arbitrary regions spread throughout the genome) except for the blood samples obtained from unrelated donors prior to their treatment in which half of the regions included in the mutagenesis panel were sequenced (24 Kbp), together with 284 exonic regions of 41 genes (73 Kbp), for a total of 97 Kbp. To exclude germline variants and somatic clonal mutations from these samples, only mutations with a variant allele frequency below 0.35 were considered. To obtain the mutation counts in the standard 96 channel profile –mutational catalog, pyrimidine perspective–, unique mutations were counted (a mutation that appears in more than 1 read is considered the same mutation originated in a divided stem cell).

Mutational signature analysis of normal tissues

The analysis of mutational signatures from bulk non-tumoral tissues sequencing data differs slightly from the analysis in tumoral tissues. In bulk tumoral tissue sequencing clonal SNVs represent a mutational snapshot of the founder cell that gave rise to the last clonal selective

sweep. Therefore counting unique SNVs provides a good representation of the trinucleotide context biases explained by the mutational processes that the founder cell was exposed to. However, in considering non-tumoral tissues we must be aware of the unequal representation of the reference trinucleotides in the bulk sequencing experiment, since the variants called do not longer represent a mutational snapshot of an individual cell, but a mixture of mutational processes drawn from a cell population.

To make the samples analyzed with both panels comparable and to conduct an unbiased mutational signature analysis we normalized the observed SNV counts per trinucleotide context in each sample by the type of panel and the sequencing depth per position. Briefly, if G_t is the genome-wide triplet content of trinucleotide t and D_t is the total sequencing depth at the sites with reference trinucleotide t in the sequencing panel (i.e. the number of times each trinucleotide had been sequenced) and $C = (C_{t>a})$ is a 96-channel profile of SNV counts with channels defined for each pyrimidine-centered trinucleotide context (with reference triplet t and alternate allele a), we normalized the profile by conducting the following rescaling componentwise $C_{t>a} \cdot G_t / D_t$. The resulting profile will be referred to as being normalized to the mappable genome triplet content and represents the expected counts of trinucleotide contexts genome wide had we sequenced the full mappable genome following the same approach as in the panel. We then used the mSigAct as described above, and given the sparsity of some of the profiles analyzed, we carried out an empirical specificity assessment to test for the significance of the presence of platinum-related mutations across samples (see below).

Mutational signature analysis in MRT-derived cell lines

The whole genome of two cell lines derived from single cells of the MRT of case 3 were sequenced. The somatic mutations in these two cell lines were identified using a regular somatic mutation calling (only Strelka2) by comparing their sequence with the matched normal blood sample. Mutations shared with the MRT sample represent clonal mutations, that is mutations present in all cells of the tumor, which are consequently expanded in both cell lines. Mutations common to both cell lines, but absent from the MRT sample are subclonal to the MRT, while mutations detected only in one cell line may be subclonal (not shared between the two selected MRT cells), private to each of the two MRT cells, or developed during their time in culture. All SNVs identified in each clone were used in the mutational signature refitting.

Mutational signature refitting

We conducted mutational signature refitting to ascertain the presence or absence of platinum related mutational signatures as plausible contributors to the mutational catalog revealed by DNA sequencing. Unlike the de novo signature extraction methods where mutational signatures are inferred from the data, signature refitting assumes that a collection of mutational signatures is given to then infer an exposure per signature and sample.

First, we selected a set of candidate, biologically plausible mutational signatures representing the common endogenous mutational processes assumed to be operative in the tissues of interest(19,20). The set includes clock-like mutational processes (SBS1, SBS5, SBS40), mutational processes related with the activity of AID/APOBEC family of cytidine deaminases (SBS2, SBS13) and reactive oxygen species induced mutagenesis (SBS18). We also include SBS45, a signature that reflects sequencing artifacts resulting from 8-oxo-guanine introduced during the sequencing protocol, which in some cases allows the necessary correction for technical noise. Finally, we include the target platinum signatures SBS31 and SBS35(21,22).

The complete set of endogenous and exogenous mutational processes is: SBS1, SBS5, SBS40, SBS2, SBS13, SBS18, SBS45, SBS31 and SBS35.

Second, considering the full set of endogenous mutational signatures alongside the exogenous target signature we implemented signature refitting as a non-negative least squares regression problem, whereby a linear model is fit with the least squares criterion and the estimated model parameters (exposures per signature per sample) are constrained to be non-negative. If S is a 96 × k matrix with the k given signature profiles arranged as columns, and M is a 96-vector of mutation counts from a sample, signature refitting finds the vector of non-negative exposures e that minimizes the reconstruction error, i.e.:

$$\hat{e} = argmin_{e \geq 0} |Se - M|^2$$

We conducted non-negative least-squares regression with all the possible combinations of up to 5 signatures against the observed mutational profiles. For each combination probed we computed the goodness of fit as an Akaike Information Criterion (AIC) score as in ref(23) which balances the reconstruction error with the complexity of the model. We deemed acceptable solutions the sets of mutational signatures yielding an AIC within a distance of 5 units or less from the minimum AIC achieved overall. We selected the most plausible mutational signatures operative in each sample based on two criteria: i) the signature set must be included among the best solution sets more frequently than any other combination with the same number of signatures; ii) the signature sets must be biologically plausible based on prior knowledge.

Third, using the most plausible endogenous mutational signatures in each tissue, we conducted a mutational signature activity test against the exogenous (platinum-related) signature with the mSigAct method. Briefly, given a set of signatures bound to explain the endogenous mutational processes (baseline), the method tests whether an additionally given target signature (platinum-related signature) significantly improves the reconstruction of the observed mutational profile. The method models the mutation count data using a negative binomial distribution and performs a likelihood ratio test comparing the likelihood of the observed catalog under two competitive models, i.e. with and without the target signature. The method yields for each sample the fitting exposures attributed to each signature (both baseline and platinum-related) alongside the significance (p-value) yielded by the statistical test.

We carried out the described analysis separately for both platinum-related signatures, SBS31 (Fig. 4 and Supplementary Fig. S12A) and SBS35 (Supplementary Fig. S12B). The SBS31 significantly increases the goodness of the fitting in samples taken after the exposure to the chemotherapy, except the MRT. This confirms the presence of platinum alterations in normal tissues. Nevertheless, in a couple of samples taken prior to the exposure to platinum, the mSigAct reconstruction shows a slight fraction of SBS31 activity (Supplementary Fig. S12A). This is the case for samples with very sparse mutational profiles, which appear negative when tested for the presence of SBS35 (also associated with the exposure to platinum-based drugs; Supplementary Fig. S12B). This prompted us to design a rigorous test for the specificity of the mSigAct assigned SBS31 activity (see below), which demonstrates that specific platinum activity is only detectable in samples taken prior to exposure to these drugs.

Platinum signatures specificity analysis

In order to detect the presence of a mutational signature in the mutational profile of a sample, the mSigAct carries out a likelihood ratio test comparing the reconstruction of the profile with and without the inclusion of the problem signature. Since we employed this test to identify the presence of the SBS31 across samples, we wanted to understand precisely its specificity,

specially for the cases with a sparse profile. To this end, we proceeded by randomly generating synthetic mutational catalogs based solely on the endogenous mutational activity of each of the samples analyzed in the manuscript. We then reran the mSigAct test on SBS31, thereby repeating the statistical test with true zero foreign exposure (i.e., true negative controls) for a number of random replicates. If the observed mutational catalog of a given sample has size N and the *k-th* endogenous signature accounts for a proportion *pk* of the total endogenous mutational exposure, we randomly draw a new mutational catalog by sampling *Nk = round(N · pk)* mutations independently from the 96-channel multinomial distribution corresponding to each *k-th* endogenous mutational signature, then adding the vectors of counts associated with all the endogenous signatures. In doing so we generated 1000 random replicates for each sample analyzed.

Finally, we run mSigAct for the SBS31 on all the random replicates and thus produced an empirical measure of the limit of detection (or specificity) as the frequency whereby the inferred foreign exposures across random replicates is equal to or higher than the observed (which could be converted into an empirical p-value). In our analyses, foreign signatures with non-zero observed levels of exposure are deemed significant only if among the 1000 random replicates there are no inferred exposures that are equal or above the observed, i.e. only if the empirical p-value is below 0.1%. This analysis showed that the exposure to SBS31 computed by the mSigAct for the EPN, MRT and blood sample from one unrelated donor were within the distribution of activities calculated for the negative control synthetic samples (Supplementary Fig. S12). This means that this calculated activity to SBS31 could be calculated from samples not really containing platinum mutations. We thus deemed the activities of SBS31 calculated by mSigAct for these samples as nonspecific (not marked with asterisk in Supplementary Fig. S11A). In coherence with this lack of specificity, no activity of SBS35, also associated with the exposure to platinum-based drugs is identified in either of these samples (Supplementary Fig. S11B).

For all other samples tested, the exposure of the true sample was at least one order of magnitude higher than the upper boundary of the distribution of negative control synthetic samples.

Inferred activity of platinum-related signatures across tumors and normal tissues

One needs to consider together the results of the signatures refitting and the specificity analysis described in the two previous sections for deep error-correcting duplex sequencing mutations, as well as the relatively shallow whole-genome sequencing mutations to make a conclusion on the activity of platinum-related signatures. In this section, we weigh all these sources of information together for the samples analyzed in this study.

The activity of SBS31 fitted by the MSigAct to the EPN sample (35 SNVs; Supplementary Fig. S11A) falls within the distribution of synthetic samples constructed in the specificity test (Supplementary Fig. S12A). It is thus not specific. The fitting with SBS35 shows no activity of this signature (Supplementary Fig. S11B), which supports the finding that platinum mutations are not present in this tumor, the sample of which was taken by surgical resection before exposure to the treatment (see case 2 clinical information). Very similar observations are made for the samples of the second tumor of cases 2 and 3 (DMG and MRT, respectively), as well as for the two blood samples taken from two unrelated donors prior to their treatment (Supplementary Fig. S11A,B and Supplementary Fig S12). Note that we did detect platinum-related mutations (SBS31) in the DMG sample in the whole-genome sequencing, which led us to the conclusion that the founder clone of this tumor was already present at the

time of exposure to the treatment for the EPN. The absence of SBS31 across low-frequency and private mutations in the DMG (with variant allele frequency below 0.35; see above) supports this conclusion. The exposure to platinum generated mutations in the founder cells of the DMG, which continued to expand for several years, before giving rise to the full blown tumor. At this stage, the platinum mutations, which were present in individual cells of the original clone, had multiplied several times through successive cell division until they reached the limit of detection of the bulk whole-genome sequencing. Finally, we hypothesize –see the main manuscript– that the lack of clonal, subclonal or private platinum-related mutations in the MRT after exposure to the treatment (Supplementary Fig. S11A,B) may be explained by the precursor cells existing in a protected niche in a (nearly) quiescent state. All samples taken from normal tissues of cases 2 and 3 show a clear signal of activity of SBS31 and SBS35 (Supplementary Fig. S11A,B and Supplementary Fig. S12), demonstrating the contribution of platinum to their mutational load.

The same process was followed to test the presence of the temozolomide-related mutational signatures in the mutational profile of the second tumor in case 3. The results of this analysis, which were negative, are presented in Figure S6A.

Somatic SNVs accumulated by the tumors in their process of clonal expansion

The clonal mutations detected in a tumor correspond to the mutations that were private in the cell that initiated the clonal expansion giving rise to the tumor. Each cell in the tumor, however, continued to accumulate SNVs throughout this process, until the tumor was clinically detected, removed, and then sequenced. In principle, the more the cells divide during this process of clonal expansion (that is, the longer the process or the more accelerated the cell cycle and proliferation), the more mutations they accumulate. Actually, the cells of both the EPN and the DMG have accumulated more subclonal and private SNVs (Supplementary Fig. S11A,B) than the number of private SNVs present in their founder cells (Fig. 2B,C).

However, the number of SNVs in one MRT cell estimated on the basis of the duplex sequencing (Supplementary Fig. S6B) is one order of magnitude smaller than the number of clonal SNVs detected in the same sample via whole genome sequencing (Supplementary Fig. S6A). This means that in the process of subclonal diversification after its most recent clonal sweep of the MRT, its average cell has accumulated fewer SNVs than the number accumulated by its most recent common ancestor in the time elapsed between the formation of the zygote and the beginning of the clonal expansion. This contrasts with what we see in the cases of the EPN and the DMG, and would suggest a slow process of subclonal diversification, with the cells of the MRT remaining in a state of (nearly) quiescence and slow growth for a period of time. This is coherent with our hypothesis to explain the complete absence of platinum-related mutations in the MRT.

Confidence intervals of inferred exposure of platinum-related signature

For samples with significant non-zero levels of exposure of the platinum-related signature we inferred confidence intervals for this exposure empirically. In a similar fashion as in the previous section, we randomly generated synthetic samples by sampling trinucleotide contexts from the 96-channel multinomial distributions given by each signature, proportionally to the total mutation burden and the proportion of exposures attributed to the signature. This time we also included mutations drawn from SBS31 at specified levels of exposure taken from a discrete grid (starting at 0 through to the total mutation burden with steps of 5 mutations). For each random replicate, the following criteria are met: i) a number of mutations (grid) are drawn from the profile of SBS31; ii) the total number of mutations equals the observed, iii) the proportions between the

endogenous mutational exposures are kept constant. With each random replicate (synthetic mutational catalog) we run the signature activity test (mSigAct) and record the inferred exposure of SBS31 alongside its significance (p-value).

As a result of this analysis, for each value of true injected SBS31 mutations (grid), we get a distribution of inferred number of mutations (n=100 replicates). The problem we address is to find a minimal (discrete) interval of true injected mutations with an overall posterior probability higher than 0.95 of producing the observed exposure.

For each value *e* of the true foreign exposure grid we fit the reconstructed exposure replicates via KDE (kernel density estimation) using a coarse bandwidth=2, yielding a probability density function ϕ_e that we can use to compute the likelihood of the observed exposure. The posterior probability that an observed exposure x resulted from a given underlying level of true exposure *e* can then be calculated via Bayes theorem as follows:

$$P(x|e) = \phi_e(x) / \sum_{j \in G} \phi_j(x)$$

where the index of the sum runs through all the values of the grid *G*.

We then ranked all the grid values (m in total) by posterior probability and selected a minimal set of top ranked exposures whose probability sum is higher than a confidence level *L*, i.e., we select $\{e_1, e_2, ..., e_s\}$ satisfying the following conditions:

$$P(x|e_1) \geq P(x|e_2) \geq ... \geq P(x|e_s) \geq ... \geq P(x|e_m)$$

$$P(x|e_1) + ... + P(x|e_{s-1}) < L$$

$$P(x|e_1) + ... + P(x|e_{s-1}) + P(x|e_s) \geq L$$

We then take the maximum and minimum values of the set as bounds for the confidence interval. In our analysis we set the confidence level at L=95% (Supplementary Fig. S14).

SNV rate per day

In order to compare the mutagenic effect of the platinum treatment with that of aging, we calculated the daily contribution of both to the SNV burden of one cell (see above). For the mutagenic effect of aging, we summarized the total exposure of the clock-like signatures (SBS1, SBS5 and SBS40); and for platinum, we took into account the SBS31 signature. We then divided the number of age-related SNVs by the age of the child at the time of the biopsy of each sample, and the number of platinum mutations by the days of platinum administration. Due to different time gaps between platinum administration in all children, we decided to count only the days in which platinum was administered to each individual. Also, it is important to take into account that platinum was administered to the four children at different doses, and with different platinum drugs: case 1 received 4 doses of carboplatin at 560 mg/m^2/day; case 2 received 16 doses of cisplatin at 30 mg/m$^{2/}$day and case 3 received 8 doses of cisplatin at 50 mg/m^2/day.

Timing the clonal expansion of a second tumor based on platinum mutations

To explain the use of platinum-related mutations to time the clonal expansion relative to the time of exposure, we can focus on two hypothetical cases represented in Supplementary Figure S2B. This simple example assumes that no reversion of mutations occurs in the progeny of the most recent common ancestor (MRCA) of this tumor in the course of its evolution. It also

assumes that the second tumor is pure and homogeneous, which is frequently the case in blood malignancies, such as t-AML. (In the case of impure tumor samples, this still applies with a simple transformation.)

In the first case, a single cell with platinum-related mutations expands into a full-blown second malignancy. As a result, all the cells in this tumor have the same platinum-related mutations, as they all derive from this MRCA of the tumor. In the second case, a clonal expansion from the MRCA of a tumor has already started at the time of exposure to the cytotoxic drug. The cells of this mass that survive the treatment (two in the toy example of the Figure) have different chemotherapy-related mutations. As these cells continue to expand (if no clonal sweep takes place), the resulting malignancy will be composed of a mixture of cells with the two different sets of mutations contributed by the two cells surviving the treatment.

If the tumor in the first case is bulk sequenced, platinum-related mutations will be distributed around a mean variant allele frequency (VAF) of 0.5 (that is, clonal mutations). This is represented in Supplementary Figure S2C (blue mutations). The randomness in the distribution of reads between the two alleles at each mutated site (the mutant and the wild-type) results in a dispersion of the VAF of treatment-related mutations around the mean value following a Poisson distribution (illustrated in Fig. S2D). In the second case, treatment-related mutations will be distributed around a mean VAF of 0.25 (subclonal orange mutations). If the expansion begins from more cells surviving the treatment, the VAF of treatment mutations is proportionally reduced (green or red mutations), until eventually, they are indistinguishable from sequencing errors (they fall below the limit of detection of bulk sequencing).

The only explanation for detecting clonal platinum-related mutations is that they were present in the MRCA of the tumor, and that the expansion from this MRCA started after the start of the exposure to the cytotoxic therapy.

Probability of KRAS mutation (tAML) generated by the platinum signature

We computed the probability that this mutation (A[C>G]C) has been generated by either SBS31 or SBS35, as representatives of the platinum exposure. To this end, we first obtained the activity of all signatures contributing to the tAML mutational profile, according to its deconstruction (see above). The signatures considered for this calculation were SBS1, SBS5, SBS40, SBS18 and either of SBS31 and SBS35. Then, we multiplied the probability of this trinucleotide context provided by the mutational profile of each of these signatures by their corresponding activity (i.e., number of mutations attributed) in the tAML. This yielded the expected number of mutations in the A[C>G]C context attributed to each signature, from which we can derive the probability that any mutation in this trinucleotide context has been generated by each signature as the proportion of expected A[C>G]C mutations that each signature contributes.

Full clinical information of the four children

Case 1

The child presented at 13 years of age with a tumor in her right lower limb. She noticed a lump in her right leg, otherwise asymptomatic. Physical exam showed a normal pubertal young girl with a hard mass palpable on her right leg. MRI showed an intra-muscle 33 mm mass with no bone involvement. The extent of disease work-up was all negative including chest CT, bone scan and bone marrow aspirates and biopsies. With a clinical suspicion of RhabdoMyosarcoma, biopsy of the tumor was performed under general anesthesia. Pathology showed *PAX3-FOXO1* rearranged Alveolar RhabdoMyosarcoma (ARMS). With the diagnosis of non-metastatic ARMS with primary tumor <5cm in the lower extremity, she was started on chemotherapy according to the MSKCC Intermediate risk protocol 2003-099A with a window phase of irinotecan and carboplatin. Disease evaluation after the window phase by right leg MRI showed a significant response of the intra-muscle tumor affecting the distal third of the vastus lateralis of 14 x 18 x 36 mm (previous 31 x 25 x 51 mm). Subsequently, the child completed planned chemotherapy and underwent second-look surgery. Pathology of the resected residual scar-looking tissue did not show evidence of tumor cells. With such complete response the child completed planned therapy with local radiation therapy (36 GY).

The child remained in complete remission until three years later when during routine follow-up, Acute Myeloblastic Leukemia (AML) (45, XX,-7,der(8)(q)[10]/ 46,XX[12]) was found. She received Fludarabine and cytarabine and reached second complete remission. Subsequently, underwent consolidation with a successful allogeneic bone marrow transplant from an HLA matched sister and the child survived.

Case 2

The child was admitted at 10 months of age, when presented with a history of 3 weeks of irritability and oriented as middle ear infection. A cranial CT was performed showing a posterior fossa tumor with calcifications causing IVth ventricle obstruction with an active supratentorial hydrocephalus. There were no familiar antecedents of neurofibromatosis or congenital bone diseases. The child had a normal milestone development until then. Physical examination showed a conscious, macro cephalic infant, with a bulging fontanel. Cervical hyperextension with neck stiffness was noted, but cervical rotation was free. No paresis in extremities were noted and cranial nerves showed no apparent involvement. No neurofibromatosis stigmata were noticed. No signs of hemi hypertrophy were seen. Cranial MRI showed a IVth ventricle tumor suggesting ependymoma. An abnormal signal affecting the left internal capsule, thalamus and left cerebral peduncle was also noticed. Spinal MRI was normal. Thoracic Xray was normal but helped disclose a left humeral bone lesion of benign radiologic characteristics. Other similar bone lesions were found, all located in left body side: vertebral left hemi hypertrophy in T7, T8 and L2 vertebra, abnormal T7 and T8 left ribs implantation, a bone lesion in the left radius, a similar bone lesion in the left femur and lastly, dysmorphic distal left tibia epiphysis. Bone scintigraphy was normal. Again, no hemi hypertrophy or neurofibromatosis stigmata were detected in reexamining the child. A ventricular-peritoneal shunt device was placed upon admission solving the neck stiffness, fontanel hypertension and irritability. Next, a macroscopically complete tumor resection was achieved. The postoperative MRI was compatible with complete tumor removal. Supratentorial mesencephalic MRI signal changes remained unmodified. Pathology was anaplastic ependymoma, WHO grade 3. Histological sections showed an ependymal cell proliferation with rosette or pseudo-rosette formation. Areas with increased cellularity were seen; these areas depicted a high proliferative index according to ki-67 IHC.

The multidisciplinary decision at the tumor board was that he needed adjuvant treatment. It was considered that radiotherapy was the best option, but given the age of the child and the anaplastic histology the treatment plan should include chemotherapy. He received adjuvant chemotherapy according to institutional protocol with irinotecan and cisplatin and then received radiation therapy in the form of protons for a total dose of 59.4 GY. He then was followed closely given the many sequelae and the bone malformations. Over the years, he developed syringomielia with tetraparesia and was not able to walk independently. Trevor syndrome was identified to explain the bone dysplasia hemi-myelica. Sequential MRIs showed residual right posterior fossa lesions that remained stable. The thickening and abnormal signal of the left internal capsule, basal ganglia, left pontine and cerebral peduncle had also remained stable. Almost nine years after the initial diagnosis, brain MRI showed a tumor centered in the right ponto-bulbar angle. The supratentorial thickening and abnormal signals remained unchanged. An open biopsy of the growing lesion was performed. Pathology reported diffuse midline H3K27 mutated glioma. With such diagnosis and the poor neurological condition, the child was managed palliatively and died of disease progression two months later. Retrospective review of the initial ependymoma tumor confirmed that the H3K27M was already present at the initial diagnosis.

Autopsy confirmed the co-existence of ependymoma and diffuse midline glioma supra and infratentorial, both associated to H3K27M. Extensive areas of diffuse K27M glioma affecting midline structures including left thalamus, left basal ganglia (areas that showed abnormal signal on MRI since initial presentation), white matter of the cerebellum, mesencephalon, pons and medulla oblongata. Also, some foci of well differentiated ependymoma were found in the left pons.

Case 3

The child presented at 17 months of age with an abdominal tumor, persistent fever, and a right eye ecchymosis together with right eye ptosis. A hard mass was palpable on the right hemi abdomen. Blood tests showed anemia (Hb 7.6 g/dL), elevated LDH (3689 UI/L), increased CRP (62.3 mg/L) and ferritin (221 ug/L). Abdominal US showed a 10 cm mass in the right hemi abdomen pushing the liver and the right kidney downwards. Head CT showed a 23 mm tumor mass in the left maxilla breaking the cortical bone and protruding into the nasal cavity and the floor of the left orbit. Chest CT was normal. Abdominal CT showed a right suprarenal mass encasing the right renal artery, celiac trunk, and superior mesenteric artery. Bone scan showed the right suprarenal mass, partially calcified, and bilateral orbit metastasis. MIBG scan showed abnormal uptakes in the right adrenal mass, bilateral orbital metastasis, and diffuse medullary uptake. Urinary catecholamines were elevated (VMA 3.6 mg/dl and HVA 36.3 mg/dL). Bone marrow aspirates showed diffuse infiltration by neuroblastoma cells; GD2 synthase highly expressed. Cardiac ECHO was normal and also audiometry. With a clinical suspicion of neuroblastoma a biopsy of the left orbit tumor mass was obtained under general anesthesia. Pathology demonstrated MYCN amplified undifferentiated neuroblastoma. With the diagnosis of stage 4 neuroblastoma, the child was enrolled on the institutional protocol(24) and received induction chemotherapy with Adriamycin, Vincristine, and Cyclophosphamide (2 cycles) alternated with (1 cycle) cisplatin and etoposide. The evaluation of response after 3 induction cycles showed complete cytological response in the 4 bone marrow aspirates and minimal residual disease (MRD) molecular testing negative, and a very good partial response (>90% shrinkage) of the retroperitoneal tumor in the MRI. After 4 cycles of induction chemotherapy, second-look surgery was performed with complete macroscopic resection of the right suprarenal tumor and regional lymph nodes. Pathology showed a good response to chemotherapy and high CHD5 expression. The end of induction evaluation (5 chemo cycles) showed complete

remission in the 4 bone marrow aspirates; MRI showed no evidence of soft tissue masses but MIBG showed persistent abnormal uptakes in both femurs and tibias. She then received 2 cycles of high dose cyclophosphamide, topotecan and vincristine. A new extent of disease evaluation showed continued complete remission in the bone marrow compartment; MIBG with lesser uptake in the right femur but persistent uptake in the left tibia. At that time the child was 23 months old, had an excellent general condition, and was referred for radiation therapy. Received 21 Gy to the primary tumor area (right adrenal gland), the distal right femur, and the left tibia. During radiation and pre/post intervals, she received cycles of irinotecan and temozolomide. A new MIBG after consolidation therapy showed disappearance of all lesions except one persistent foci in the left tibia. Bone marrow studies remained negative as well as urinary catecholamines. A CT of the left tibia did not show detectable abnormality. MIBG guided surgical removal of the tibia lesion was performed and histology confirmed chemorefractory undifferentiated neuroblastoma residing in the cortical bone. A confirmatory MIBG was performed after which finally became completely negative 11 months after first diagnosis. Once complete remission status was achieved, 2 cycles of high-dose cyclophosphamide and irinotecan were administered before mu3F8 immunotherapy was administered at MSKCC, NY. A new extent of disease work-up including MIBG, CT, and bone marrow studies showed persistent complete remission. She received 5 planned cycles of mu3F8 based immunotherapy including cyclophosphamide and rituximab because HAMAs became positive after the second cycle. She completed all planned therapy 20 months after initial diagnosis and underwent close follow-up since then, showing normal growth and development along with continued complete remission until 8.5 years later.

The child was 10 years of age when presented with asthenia, loss of appetite and fatigue. She had left flank pain but no bone pain or fever. Abdominal US suggested a large mass and abdominal MRI showed a well-defined mass in the left suprarrenal region 2.7 x 3.5 x 4.5 cm, T2 hyperintense, and heterogeneous in its diffusion restriction. Lymph nodes of the ipsilateral para aortic chain above and below the left kidney vascular pedicle were enlarged suggesting lymph node involvement. Right kidney was smaller than the left because of prior radiotherapy. Liver, spleen, and all the other abdominal organs were normal. With a clear suspicion of neuroblastoma relapse, full disease work-up was performed including MIBG and bone marrow studies. MIBG and BM studies were negative. Surgery was then performed with left adrenalectomy and gross total resection of the retroperitoneal mass. Surgically resected mass was 5 x 3.4 x 4 cm and 33 grams and the adjacent left adrenal gland 6.5 x 1.3 cm. Histopathology described a diffuse cellular proliferation including nested and trabeculated areas surrounded by fibrous septae. Neoplastic cells were large, with pleomorphic nuclei, vesicular chromatin, and occasional prominent nucleoli. These cells did not look like neuroblastoma cells. Some foci of desmoplastic fibrosis were identified as well as hemorrhagia without clear necrosis. There was vascular and perineurial invasion. The tumor was infiltrating the adrenal gland but was not arising from it. Immunohistochemical profile of the tumor cells showed positive staining for CK8/18, EMA, AE1/AE3, vimentin, CK7, and CK20 and were negative for synaptophisin, chromogranin, NB84, S100, alpha-inhibin, HMB45, myogenin, myo-D1, and nuclear beta-catenin. p53 was expressed patchy. Ki67 was 30%. INI-1 was not expressed. *SMARCB1* mutation was confirmed in the tumor by NGS showing p.V270fs along with a large deletion in the other allele. The diagnosis of malignant rhabdoid tumor was further confirmed by methylome. The MRT showed 25-30% expression of PD-L1 and 25-30% of CD4 and CD8 cells in the periphery of the tumor. Extent of disease work-up by PET-FDG (8/4/2019) showed abnormal uptake in the retroperitoneal mass (SUV 4.7), bilateral lung nodules mainly in the left (SUV 5.1) and 2 bone lesions in the head of the right humerus (SUV 3.2) and L5 vertebral body (SUV 5.9). Given the diagnosis of metastatic soft tissue malignant RT and the virtually incurable prognosis, the decision was to target treatment with 2 cycles of the anti-PD1 atezolizumab but

disease progressed. Given the expression of SPARC, nab-paclitaxel (AbraxaneR) was added to atezolizumab but disease continued to progress. She was sent to palliative care and died of progressive disease 6 months after the diagnosis of MRT and 9 years after the diagnosis of NB. Parents granted an autopsy and all tissues were preserved immediately through snap freezing.

Case 4

The child presented at 4 years of age with a tumor in her right mandible and associated cervical lymph nodes. She noticed a lump in her right mandible, with low grade temperature and malaise. Physical exam showed a normal prepubertal girl with a hard mass palpable on her right cervical region and mandible. MRI showed a bone infiltrating soft tissue mass with regional cervical lymph nodes enlarged up to a maximum diameter of 46 mm. Abdominal ultrasound revealed disseminated enlarged lymph nodes affecting the pancreas, both kidneys and the ovaries bilateral. FDG-PET showed the abnormal uptake of the disseminated lymph nodes and the jaw-associated tumor mass suggesting of lymphoproliferative disease. Bone scan confirmed the bone lesion in the right jaw. Biopsy of the cervical lymph nodes confirmed Burkitt's Lymphoma (BL). With the diagnosis of stage 4 –metastatic to bone- BL, she was started on chemotherapy according to the International NHL B 2004 protocol with rituximab, treatment group BII because of negative FDG-PET after the COPADM2 cycle. She completed treatment uneventfully and entered complete remission right after induction and remained in complete remission ever since. No surgery or radiotherapy were administered. She developed well and 8 years later developed puberty normally with no hormonal deficits.

Being asymptomatic, 11 years after first diagnosis and at age 15, a thyroid nodule was detected by physical exam. As familial antecedents, mother is affected of Hashimoto thyroiditis. Serial thyroid ultrasound studies confirmed a poorly defined nodule, hyper vascularized, with associated micro calcifications, 7 x 6 x 4 mm. Total thyroidectomy was eventually performed confirming the suspicion of papillary thyroid carcinoma with no evidence of lymph node invasion and clear margins. Follow-up has not revealed metastatic disease and she remains in continued complete remission of both neoplasia.

References

1. Benjamin D, Sato T, Cibulskis K, Getz G, Stewart C, Lichtenstein L. Calling Somatic SNVs and Indels with Mutect2. bioRxiv. Cold Spring Harbor Laboratory; 2019;861054.
2. Kim S, Scheffler K, Halpern AL, Bekritsky MA, Noh E, Källberg M, et al. Strelka2: fast and accurate calling of germline and somatic variants. Nat Methods. 2018;15:591–4.
3. Chen S, Francioli LC, Goodrich JK, Collins RL, Kanai M, Wang Q, et al. A genome-wide mutational constraint map quantified from variation in 76,156 human genomes [Internet]. bioRxiv; 2022 [cited 2023 Jun 6]. page 2022.03.20.485034. Available from: https://www.biorxiv.org/content/10.1101/2022.03.20.485034v2
4. Poplin R, Ruano-Rubio V, DePristo MA, Fennell TJ, Carneiro MO, Auwera GAV der, et al. Scaling accurate genetic variant discovery to tens of thousands of samples [Internet]. bioRxiv; 2018 [cited 2023 Jun 6]. page 201178. Available from: https://www.biorxiv.org/content/10.1101/201178v3
5. Priestley P, Baber J, Lolkema MP, Steeghs N, de Bruijn E, Shale C, et al. Pan-cancer whole-genome analyses of metastatic solid tumours. Nature. 2019;575:210–6.
6. Martínez-Jiménez F, Movasati A, Brunner S, Nguyen L, Priestley P, Cuppen E, et al. Pan-cancer whole genome comparison of primary and metastatic solid tumors [Internet]. bioRxiv; 2022 [cited 2023 Feb 15]. page 2022.06.17.496528. Available from: https://www.biorxiv.org/content/10.1101/2022.06.17.496528v1
7. Ng AWT, Poon SL, Huang MN, Lim JQ, Boot A, Yu W, et al. Aristolochic acids and their derivatives are widely implicated in liver cancers in Taiwan and throughout Asia. Sci Transl Med. 2017;9.
8. McLaren W, Gil L, Hunt SE, Riat HS, Ritchie GRS, Thormann A, et al. The Ensembl Variant Effect Predictor. Genome Biol. 2016;17:122.
9. Rahman N. Realising the Promise of Cancer Predisposition Genes. Nature. 2014;505:302–8.
10. Fiala EM, Jayakumaran G, Mauguen A, Kennedy JA, Bouvier N, Kemel Y, et al. Prospective pan-cancer germline testing using MSK-IMPACT informs clinical translation in 751 patients with pediatric solid tumors. Nat Cancer. Nature Publishing Group; 2021;2:357–65.
11. Akhavanfard S, Padmanabhan R, Yehia L, Cheng F, Eng C. Comprehensive germline genomic profiles of children, adolescents and young adults with solid tumors. Nat Commun. Nature Publishing Group; 2020;11:2206.
12. Cameron DL, Schröder J, Penington JS, Do H, Molania R, Dobrovic A, et al. GRIDSS: sensitive and specific genomic rearrangement detection using positional de Bruijn graph assembly. Genome Res. 2017;27:2050–60.
13. Cameron DL, Baber J, Shale C, Valle-Inclan JE, Besselink N, van Hoeck A, et al. GRIDSS2: comprehensive characterisation of somatic structural variation using single breakend variants and structural variant phasing. Genome Biol. 2021;22:202.
14. Cibulskis K, Lawrence MS, Carter SL, Sivachenko A, Jaffe D, Sougnez C, et al. Sensitive detection of somatic point mutations in impure and heterogeneous cancer samples. Nat Biotechnol. Nature Publishing Group; 2013;31:213–9.
15. Coorens THH, Farndon SJ, Mitchell TJ, Jain N, Lee S, Hubank M, et al. Lineage-Independent Tumors in Bilateral Neuroblastoma. N Engl J Med. Massachusetts Medical Society; 2020;383:1860–5.
16. Danecek P, Bonfield JK, Liddle J, Marshall J, Ohan V, Pollard MO, et al. Twelve years of SAMtools and BCFtools. GigaScience. 2021;10:giab008.
17. Danecek P, Auton A, Abecasis G, Albers CA, Banks E, DePristo MA, et al. The variant call format and VCFtools. Bioinformatics. 2011;27:2156–8.
18. Robinson JT, Thorvaldsdóttir H, Winckler W, Guttman M, Lander ES, Getz G, et al.

Integrative genomics viewer. Nat Biotechnol. Nature Publishing Group; 2011;29:24–6.
19. Li R, Di L, Li J, Fan W, Liu Y, Guo W, et al. A body map of somatic mutagenesis in morphologically normal human tissues. Nature. Nature Publishing Group; 2021;597:398–403.
20. Moore L, Cagan A, Coorens THH, Neville MDC, Sanghvi R, Sanders MA, et al. The mutational landscape of human somatic and germline cells. Nature. Nature Publishing Group; 2021;597:381–6.
21. Alexandrov LB, Kim J, Haradhvala NJ, Huang MN, Tian Ng AW, Wu Y, et al. The repertoire of mutational signatures in human cancer. Nature. 2020;578:94–101.
22. Pich O, Muiños F, Lolkema MP, Steeghs N, Gonzalez-Perez A, Lopez-Bigas N. The mutational footprints of cancer therapies. Nat Genet. 2019;51:1732–40.
23. Rahbari R, Wuster A, Lindsay SJ, Hardwick RJ, Alexandrov LB, Al Turki S, et al. Timing, rates and spectra of human germline mutation. Nat Genet. Nature Publishing Group; 2016;48:126–33.
24. Mora J, Cruz O, Lavarino C, Rios J, Vancells M, Parareda A, et al. Results of induction chemotherapy in children older than 18 months with stage-4 neuroblastoma treated with an adaptive-to-response modified N7 protocol (mN7). Clin Transl Oncol. 2015;17:521–9.
