# Supplementary material for: Origins of second tumors in children and mutational footprint of chemotherapy in normal tissues
Source: Cancer Discov. Author manuscript; Available in PMC 2024 Jun 4. (PMC11145171; doi:10.1158/2159-8290.CD-23-1186)
Supplement: Table S1 [file EMS194327-supplement-Table_S1.pdf]

Supplementary Table 1

Case	Gene	Role in cancer	Variant type	Amino acid change	gnomADg Allele Frequency
**1**	ATM	LoF	Missense	V182L	0.007418
**1**	ATM	LoF	Missense	R2854C	0.000126
**1**	EGFR	Act	Missense	A1210V	0.000328
**1**	MET	Act	Splice region variant	-	0.001314
**1**	CBL	Act	Missense	P687L	0.000119
**1**	ITK	NA	Missense	R193Q	0.003875
**1**	EP300	LoF	Missense	G211S	0.006329
**1**	FAT1	LoF	Missense	F2549L	0.000223
**1**	FANCM	NA	Missense	D556G	0.000105
**1**	JMJD1C	NA	Missense	S1429L	0.000545
**1**	NDRG4	NA	Splice region variant	-	0.000077
**2**	DICER1	LoF	Missense	Y1385C	0
**2**	SERPINA1	NA	Frameshift	E347X	0.000253
**2**	WRN	LoF	Missense	T1262R	0.002702
**2**	CFTR	NA	Missense	G576A	0.005081
**2**	CFTR	NA	Missense	R668C	0.006121
**3**	RAF1	Act	Splice region variant	H389	0.000063
**3**	RAD50	NA	Missense	Q426R	0.000147
**3**	CDH1	LoF	Missense	P30T	0.001278
**3**	COL7A1	NA	Missense	G636V	0.004155
**3**	RTEL1	NA	Inframe	GE770-771G	0.004962
**3**	GBA	NA	MIssense	T408M	0.006204
**3**	SLX4	NA	Missense	R237Q	0.008306
**4**	PTCH1	LoF	MIssense	D436N	0.000824
**4**	SDHD	LoF	MIssense	G12S	0.006979
**4**	SDHB	LoF	MIssense	G53E	0.000405
**4**	MET	Act	MIssense	T1010I	0.00897
**4**	PDGFRA	Act	MIssense	G79D	0.008872
**4**	WAS	Act	MIssense	V332A	0.004856
**4**	SERPINA1	NA	Frameshift	E347X	0.000253
**4**	AR	Act	Inframe	GGGGG457-461-	0.009286
**4**	JMJD1C	NA	MIssense	F130Y	0.000049
**4**	SHOC2	NA	MIssense	E25G	0.000077

Supplementary Table 1. Germline SNVs and indels found in known cancer predisposing genes.
Gene: HGNC Symbol; Role in cancer: Loss of Function (LoF), NA (Not known, ambiguous); Variant type: Missense, Frameshift (truncating), Splice region variant, Inframe; Amino acid change: Reference amino acid + protein position + mutated amino acid; gnomADg Allele Frequency: allele frequency of the mutation in gnomADg population. No clear predisposing variant is present in any child.
